# Extracellular matrix stiffness regulates colorectal cancer progression via HSF4

**DOI:** 10.1186/s13046-025-03297-8

**Published:** 2025-01-30

**Authors:** Kangtao Wang, Siyi Ning, Shuai Zhang, Mingming Jiang, Yan Huang, Haiping Pei, Ming Li, Fengbo Tan

**Affiliations:** 1 Department of General Surgery, Xiangya Hospital, Central South University, Changsha, Hunan 410008 China; 2 Department of General, Visceral & Transplant Surgery, Molecular OncoSurgery, Section Surgical Research, University of Heidelberg, Heidelberg, Baden-Württemberg 69117 Germany; 3 Clinical Laboratory, Changsha Stomatology Hospital, Changsha, Hunan 410005 China; 4 Department of Ultrasonography, Xiangya Hospital, Central South University, Changsha, Hunan 410008 People’s Republic of China; 5 NHC Key Laboratory of Birth Defect for Research and Prevention, Hunan Provincial Maternal and Child Health Care Hospital, Changsha, Hunan 410008 China; 6 Hunan Provincial Key Laboratory of Neurorestoration, Changsha, Hunan 410081 China; 7 Department of Immunology, College of Basic Medical Sciences, Central South University, Changsha, Hunan 410008 China; 8 National Clinical Research Center for Geriatric Disorders (Xiangya Hospital), Central South University, Changsha, Hunan 410008 China; 9 The “Double-First Class” Application Characteristic Discipline of Hunan Province (Clinical Medicine), Changsha Medical University, Changsha, Hunan 410219 China

**Keywords:** Colorectal cancer, Tumour stiffness, Magnetic resonance elastography, Heat shock transcriptional factor 4, Extracellular matrix, Transcriptome sequencing

## Abstract

**Background:**

Colorectal cancer (CRC) has high incidence and mortality rates, with severe prognoses during invasion and metastasis stages. Despite advancements in diagnostic and therapeutic technologies, the impact of the tumour microenvironment, particularly extracellular matrix (ECM) stiffness, on CRC progression and metastasis is not fully understood.

**Methods:**

This study included 107 CRC patients. Tumour stiffness was assessed using magnetic resonance elastography (MRE), and collagen ratio was analysed with Masson staining. CRC cell lines were cultured on matrices of varying stiffness, followed by transcriptome sequencing to identify stiffness-related genes. An HSF4 knockout CRC cell model was cultured in different ECM stiffness to evaluate the effects of HSF4 on cell proliferation, migration, and invasion in vitro and in vivo.

**Results:**

CRC tumour stiffness was significantly higher than normal tissue and positively correlated with collagen content and TNM staging. High-stiffness matrices significantly regulated cell functions and signalling pathways. High HSF4 (heat shock transcriptional factor 4) expression was strongly associated with tumour stiffness and poor prognosis. HSF4 expression increased with higher TNM stages, and its knockout significantly inhibited cell proliferation, migration, and invasion, especially on high-stiffness matrices. In vivo experiments confirmed that HSF4 promoted tumour growth and metastasis, independent of collagen protein increase.

**Conclusions:**

This study reveals that tumour stiffness promotes the proliferation and metastasis of CRC by regulating EMT-related signalling pathways through HSF4. Tumour stiffness and HSF4 could be valuable targets for prognostic assessment and therapeutic intervention in CRC.

**Supplementary Information:**

The online version contains supplementary material available at 10.1186/s13046-025-03297-8.

## Introduction

Colorectal cancer (CRC) continues to pose a significant global health challenge, ranking as the third most common cancer with over a million new cases annually [1]. Despite notable advancements in early screening technologies, surgical techniques, and adjuvant therapies, the prognosis for CRC patients, particularly those in advanced stages, remains fraught with uncertainties [2, 3]. In recent years, tumour stiffness has emerged as a novel biomarker garnering significant scientific attention for its potential in predicting CRC prognosis [4–8].

Tumour stiffness, a physical characteristic, profoundly influences the biological behaviour of tumours [9]. It reflects the complex composition and refined structure of the extracellular matrix (ECM) resulting from the intricate dynamic interactions between cells and the ECM [10]. The ECM, acting as a microecosystem for cancer cells, comprises various components, such as collagen content, fibre alignment, and cross-linking strength, all of which contribute to the unique stiffness profile of the tumour [11]. Research has shown that ECM stiffness can translate physical stimuli into biochemical signals through precise mechanotransduction mechanisms, thereby regulating gene expression, cell proliferation, migration, and invasion [12]. This process reshapes the tumour microenvironment and drives the evolution of the tumour towards a malignant phenotype [13]. However, the specific mechanisms, key genes involved, and the role of collagen in this process remain unclear.

The heat shock transcriptional factor (HSF) family, a key player in transcriptional regulation, is indispensable in cellular stress response [14, 15]. These factors are activated in response to various stress signals, regulating the expression of heat shock proteins (HSPs) and other stress-related genes to help cells maintain homeostasis [16]. Recent in-depth explorations have unveiled broader biological functions of HSFs, particularly their critical roles in ECM remodelling and tumour progression [17]. HSFs may influence the expression of ECM-related genes directly or indirectly, adjusting ECM composition and stiffness and thus acting as significant drivers in tumour development [18]. However, the specific mechanisms of the HSF family in the context of CRC and their intrinsic links to tumour stiffness remain fully elucidated, presenting a broad avenue for research.

In this study, we employed a range of research methods, including magnetic resonance elastography (MRE), transcriptome sequencing, in vitro experiments, in vivo animal models, and clinical data analysis, to elucidate the mechanism by which stiffness promotes CRC invasion and metastasis through HSF4, leading to poor prognosis. Our findings indicate that HSF4 expression levels are closely associated with tumour stiffness and positively correlated with poor prognosis in CRC patients. Further analysis revealed that HSF4 enhances CRC invasion and metastasis via the epithelial-mesenchymal transition (EMT) signalling pathway, independent of changes in collagen content. These discoveries underscore the central role of the mechanical properties of the tumour microenvironment in CRC progression and highlight the potential of HSF4 as a therapeutic target, paving the way for new strategies in CRC prevention, diagnosis, and treatment.

## Materials and methods

### Study design and participants

Our prospective study (ID: 201,903,078) was approved by the Institutional Review Board, and all participants provided written informed consent. We collected clinical samples from CRC patients who underwent comprehensive evaluation at Xiangya Hospital, Central South University, China. Treatment plans were determined through discussions with the multidisciplinary team (MDT). This study did not influence the treatment choice; patients received the best treatment based on MDT evaluations. Recruitment took place from December 2018 to December 2019, with follow-up continuing until December 2023, resulting in an average follow-up period of 58 months. Samples were collected during surgery or biopsy, following standard laboratory practice guidelines. The hospital's internal review board and ethics committee reviewed and approved all sample collection and preservation procedures.

### MRI image acquisition and MR elastography

MRI and MRE were conducted using a sequence and setup similar to our previous research [19, 20]. All patients began a liquid diet one day before their MRI scan and adhered to a strict 4-h fasting protocol before imaging. The MRI scans were performed using an 18-channel phased-array body coil on a 3 T scanner (Magnetom Prisma, Siemens Healthcare, Germany). Conventional T2-weighted (T2w) images of the rectum were acquired using 2D fast spin-echo (FSE) sequences in oblique, sagittal, and coronal planes, with an in-plane resolution of 3 × 3 mm. Additionally, 3D FSE (SPACE) T2w images were obtained with an in-plane resolution of 0.8 × 0.8 mm^2^. The total time to acquire the anatomical images was 12 min.

As for MRE, Mechanical waves at 40, 50, 60, and 70 Hz vibration frequencies were delivered to the pelvic region using three surface-based pneumatic actuators. Two actuators were positioned at the posterior region with a static pressure of 0.8 bar, while one actuator was placed at the anterior pelvic region, specifically at the top of the pubic symphysis, with a static pressure of 0.7 bar. A single-shot spin-echo echo-planar imaging (SE-EPI) sequence with motion-encoded gradients (MEG) for flow compensation was used to capture a complete 3D wave field. The entire vibration cycle was sampled at eight phase offsets. Continuous acquisition of fifteen 5 mm thick sagittal slices with a resolution of 3 × 3 mm^2^ was performed during free-breathing. MRE frequencies were optimised for each vibration frequency (40, 50, 60, and 70 Hz) and set at 47.89, 47.89, 47.89, and 52.41 Hz, respectively. Additional imaging parameters included an echo time of 56 ms, a repetition time of 1670 ms, a GRAPPA factor of 2 for parallel imaging, and a MEG amplitude of 50 mT/m. The total acquisition time was 3.5 min.

### Image analysis

After imaging, a radiologist with 5 years of experience in gastrointestinal imaging assessed the tumour location, TNM staging, and other relevant details on T2w images using the DISTANCE method. This method provides a systematic approach for evaluating all clinically significant features on MR images, which is crucial for treatment planning. In the DISTANCE method: DIS stands for the distance from the inferior part of the tumour to the transitional skin; T stands for T staging; A stands for the anal complex; N stands for nodal staging; C stands for circumferential resection margin; and E stands for extramural vascular invasion. MRI-based T and N staging were assigned to each patient using this method.

The analysis method for MRE datasets was detailed in previous studies. In brief, the MRE data was processed using wave-number multifrequency-inversion (k-MDEV) to create parameter maps of shear wave speed $\backslash(c\backslash)$ (measured in m/s), which serves as a proxy for tissue stiffness. The data processing followed the k-MDEV pipeline, which is available at www.bioqic-apps.com. To characterise the tumour, 9 to 18 circular regions of interest (ROIs), each measuring 0.3 ± 0.02 cm^2^, were placed in the anterior and posterior rectal wall across three consecutive slices covering the most significant solid tumour cross-section, based on anatomical T2w images and avoiding areas of necrosis, cyanosis, and blood vessels. The stiffness values were averaged within these manually defined ROIs. Distal tumour-adjacent tissue (DTT), located 2 cm from the tumour, was analysed in 6 circular ROIs measuring 0.1 ± 0.02 cm^2^ as a reference. In order to avoid the influence of individual differences in tissue stiffness on tumour stiffness, tumour stiffness was calculated as the ratio of the average shear wave velocity in tumour tissue to the average shear wave velocity in normal tissue. A radiologist, blinded to the clinical outcomes, placed all ROIs using MRE magnitude images and the corresponding elastograms.

### Immunohistochemistry (IHC) and masson staining

Immunohistochemistry was performed on human colorectal cancer tissues and tumour and liver metastasis tissues from nude mice. The detection of Lysyl Oxidase-Like 1 (LOXL1), HSF4, and Alpha-Smooth Muscle Actin (αSMA) followed a standardised protocol. Paraffin-embedded sections were first deparaffinised and rehydrated using an eco-friendly deparaffinisation solution (Servicebio, G1128) and anhydrous ethanol (SCRC, 100,092,683), followed by thorough rinsing with distilled water to ensure complete removal of residual chemicals. Antigen retrieval was subsequently performed using a 20 × Citrate Antigen Retrieval Solution (pH 6.0) (Servicebio, G1202). The specific retrieval conditions for each antigen were optimised based on tissue type and antigen characteristics, with careful attention to prevent buffer evaporation during the retrieval process. For all targets (LOXL1, HSF4, and α-SMA), antigen retrieval involved microwave heating at medium heat for 8 min, followed by an 8-min pause and then continued with low-medium heat for 7 min. After retrieval, the slides were washed with PBS (Servicebio, G0002), and endogenous peroxidase was exhausted by incubating the sections in a 3% hydrogen peroxide solution (Angergech, 04008978274207), followed by another PBS wash. Serum blocking was performed by covering the tissue with 3% BSA (Servicebio, GC305010) at room temperature. The primary antibody, prepared in the appropriate dilution, was applied to the sections and incubated overnight at 4 °C. The secondary antibody, HRP-labeled and corresponding to the species of the primary antibody, was then applied and incubated at room temperature. DAB (Servicebio, G1212) staining was performed next, with controlled development time, followed by a rinse with tap water to stop the reaction. Nuclei were counterstained, and the sections were dehydrated and mounted (Servicebio, G1404-100 mL). The results were examined under a bright-field microscope for LOXL1 and α-SMA; nuclei stained blue with hematoxylin (Servicebio, G1039), while positive expression indicated by DAB appeared brownish-yellow. HSF4 immunohistochemical staining intensity was classified into four levels: 0 (negative), 1 (weak), 2 (moderate), and 3 (strong). Staining frequency was quantified using ImageJ software through pixel analysis, with manual threshold adjustments based on each image's background noise and staining characteristics. To minimise bias, we performed multiple repeated observations and image comparison analyses. In the frequency quantification, the denominator included only tumour region pixels, excluding background noise and non-tumour tissues. The final HSF4 score was calculated by multiplying staining intensity by staining frequency, where a score > 1 was classified as high expression and a score of 1 or less as low expression. The HSF4 scoring criteria were referenced from Zhang et al.'s study [21]. These methods aim to enhance the quantitative analysis's transparency and reliability, ensuring the results' accuracy and reproducibility.

Masson staining is a widely used histochemical technique highlighting structures such as collagen and muscle fibres within tissues. For this study, sections were processed according to the manufacturer's instructions (Servicebio) for Masson staining. The procedure involved deparaffinisation and rehydration of paraffin-embedded sections or warming and fixing frozen sections, followed by sequential staining with the provided solutions. After staining, the sections were differentiated in 1% acetic acid, dehydrated, cleared, and mounted with neutral gum. Under the microscope, collagen fibres appeared blue, while muscle fibres, fibrin, and red blood cells were stained red. Details of the specific antibodies and staining kit used are provided in Table S1.

### Cell culture and cell transfection

Two human colorectal cancer cell lines, SW480 and HCT116, and a colon fibroblast cell line, CCD-18Co, were obtained from the American Type Culture Collection. SW480 cells were maintained in Leibovitz's L-15 medium (Gibco) with 10% fetal bovine serum (FBS) (Hyclone, Logan, UT, USA), 2 mM L-glutamine, 0.1 mg/mL streptomycin, and 100U/mL penicillin at 37 °C in a standard humidity incubator. HCT116 cells were cultured in RPMI 1640 medium (Invitrogen) with 10% FBS and maintained in a humidified incubator at 37 °C with 5% CO_2_. CCD-18Co cells were cultured in Dulbecco's Modified Eagle Medium (DMEM) with high glucose (Life Technology, NY, USA), supplemented with 100 mg/ml streptomycin, 100 units/ml penicillin, and 10% fetal bovine serum. The cells were maintained at 37 °C with 5% CO_2_ and ≥ 90% humidity.

Genchem Biotechnology Co. Ltd. (Shanghai) provided lentiviral-based small hairpin RNA (shRNA) targeting HSF4 and a control lentivirus with scrambled shRNA. The sequences used were si-HSF4 #1: 5’-GCAAGCUGAUCCAGUGUCUTT-3', si-HSF4 #2: 5’-CGCCAACUCAACAUGUACGTT-3', and scrambled: 5’-UUCUCCGAACGUGUCACGU-3'. Experiments found that si-HSF4 #2 was more effective than si-HSF4 #1; therefore, only si-HSF4 #2 was used for shRNA constructs. SW480 and HCT116 cells were infected with these lentiviral particles and maintained in L-15 or RPMI 1640 medium containing 2 μg/mL or 1 μg/mL puromycin, respectively. After two weeks of selection, western blot analysis was performed to measure HSF4 expression, confirming the creation of stable cell lines.

### Construction of different matrix stiffness models

The construction of different matrix stiffness models followed established protocols reported in the literature [22]. Soft and stiff matrix media were obtained from MATRIGEN, USA (CAT SW6-COL-2-EA). The parameters for the stiff matrix media were 25 kPa and 50 hydrogels coated in different numbers of healthy plates. The parameters for the soft matrix media were 2 kPa hydrogels coated in different numbers of well plates.

### RNA extraction and deep sequencing

For RNA extraction and deep sequencing, SW480 cells were cultured on 6-well plates coated with 2 kPa or 25 kPa hydrogels (MATRIGEN, USA). Plates were washed with PBS, and 2 ml of cell suspension at 1 × 10^6^ cells/ml was added to each well. Each stiffness condition was tested in triplicate, and cells were incubated at 37 °C in a CO_2_-free incubator for 72 h. Cells were harvested by scraping in a complete medium. The cell suspension was centrifuged, washed with PBS, and treated with TRIzol reagent (Life Technologies) for RNA extraction, which was then sent to BGI Genomics for processing. BGI extracted and reverse-transcribed it into cDNA and constructed libraries for deep sequencing with a depth of 200 × . RNA was quantified using a NanoDrop and Agilent 2100 bioanalyser (Thermo Fisher Scientific). Libraries were prepared from 1 μg of total RNA per sample, and small RNA (18–30 nt) was purified via PAGE gel electrophoresis. Adapter-ligated small RNA was reverse-transcribed into cDNA, amplified by PCR, and enriched cDNA fragments were selected and purified. Library quality and quantification were assessed using an Agilent 2100 bioanalyser and real-time qPCR with TaqMan probes. Sequencing was performed on the Illumina HiSeq platform (BGI, Shenzhen). All sequencing results were uploaded to the Sequence Read Archive (SRA) database in FASTQ format. A total of six files were submitted, with each specimen generating an average of 12.64 GB of data. The dataset can be accessed at (https://www.ncbi.nlm.nih.gov/bioproject/PRJNA522032).

### Sequencing data processing, differentially expressed genes and functional analysis

FASTQ files were subjected to quality assessment and potential coding predictions. The reads were then normalised to FPKM (Fragments Per Kilobase of exon model per Million mapped reads) to create matrix files. The processed data have been uploaded to the GEO database under accession number GSE273846 (https://www.ncbi.nlm.nih.gov/geo/query/acc.cgi?acc=GSE273846). Differential expression analysis was performed in R using the DESeq2 package, which involved normalising counts, estimating dispersion, and fitting a generalised linear model to identify differentially expressed genes (DEGs) [23]. Gene Ontology (GO) and pathway enrichment analyses were conducted using the clusterProfiler package in R to elucidate the biological significance of the DEGs. Additionally, single-sample gene set enrichment analysis (ssGSEA) was carried out with the GSVA package in R to assess pathway activity at the individual sample level. Statistical analyses and visualisations, including plots of DEGs, enriched GO terms, and pathway activities, were generated using R packages such as ggplot2 [24]. Appropriate corrections for multiple tests were applied to ensure the robustness of the results. DEGs were visualised using volcano plots and heat maps to effectively illustrate the differences and patterns in gene expression. Please refer to our previous publication for detailed analysis methods [24].

### Data sources and analysis for HSF4 mRNA expression in CRC patients

HSF4 mRNA expression data and clinical information for CRC patients were retrieved from The Cancer Genome Atlas (TCGA) and the GSE10950 datasets [25]. The TCGA CRC cohort included 41 matched pairs of CRC tumours and adjacent normal tissues and 458 unpaired CRC tumour samples. TCGA-related analyses and visualisations were conducted using the GEPIA2 database (http://gepia2.cancer-pku.cn/) [26].

### Cell proliferation assay using CCK-8

In order to evaluate the effect of HSF4 knockdown on tumour cell proliferation, a CCK-8 assay was conducted. The cells were seeded at a density of 2 × 10^4^ cells per well in a 96-well plate containing 100 μl of medium with varying stiffness (2 kPa or 25 kPa). After incubation under standard conditions, the viability of the cells was assessed each day using the CCK-8 reagent. Viable cells were incubated with the CCK-8 solution for 2 h, and the absorbance at 450 nm was measured. This absorbance provided a quantitative measure of cell proliferation, allowing for the assessment of the impact of HSF4 knockdown on the growth of HCT116 and SW480 cells.

### Transwell migration and matrigel invasion assays

Transwell migration and Matrigel invasion assays were conducted using 24-well culture plates with inserts containing 8 μm pore membranes (Falcon). These membranes were either uncoated or coated with Matrigel (Corning). The cells were cultured on 2 kPa or 25 kPa matrices for 48 h, followed by starvation for 24 h before being harvested and seeded into the chambers for migration and invasion tests. 4 × 10^4^ cells in 0.2% bovine serum albumin (BSA) were placed in the upper chamber, while the lower chamber was filled with 500 μL of medium containing 10% fetal bovine serum (FBS). After 24 h, the cells that had migrated to the bottom surface of the upper chamber were fixed with 4% paraformaldehyde for 20 min and then stained with 0.1% Crystal Violet. The migrated or invaded cells were counted and photographed using a microscope, with five fields per membrane analysed for each group. These experiments were performed in triplicate. For a more detailed description of this methodology, please refer to our previous publication [27].

### Wound healing assay

Cells from each group were trypsinised and seeded into 6-well plates, then incubated at 37 °C until they reached 90% confluence. The cells were subjected to serum starvation in a 0.1% FCS-medium for 24 h. A precise scratch was made across the cell monolayer using a 10 µL pipette tip. After scratching, the cells were gently rinsed with PBS and cultured in 2 mL of 1% FCS-medium. The initial gap width was recorded at 0 h using a Leica microscope. The gap was then re-measured at 24 and 48 h with Image J software, and the percentage of the remaining gap area was calculated.

### Western Blot (WB) analysis

The Western blot analysis was conducted according to the methodology described in our previous research [28]. Briefly, cells were lysed in RIPA buffer containing a protease inhibitor cocktail (Selleck). Proteins (30 μg) were denatured by boiling at 95 °C for 5 min and then separated using sodium dodecyl sulfate–polyacrylamide gel electrophoresis (SDS-PAGE). The separated proteins were transferred to a PVDF membrane, blocked with 5% non-fat milk in TBS buffer containing 0.1% Tween-20 (TBS-T) at room temperature for 1 h. The membranes were cut according to molecular weights, incubated with primary antibodies at 4 °C overnight, washed with TBS-T, and then treated with HRP-conjugated secondary antibodies for 2 h at room temperature. Signal detection was performed using an enhanced chemiluminescence (ECL) system (Amersham Pharmacia Biotech, Arlington Heights, IL). Each blot was performed in triplicate, with GAPDH as the loading control. The intensity of the bands was measured using ImageJ for relative quantification. The Epithelial-Mesenchymal Transition (EMT) Antibody Sampler Kit #9782 was used to analyse epithelial-mesenchymal transition. The specific antibodies used are detailed in Table S1.

### Immunofluorescence assay

Immunofluorescence assays were performed using the CST immunofluorescence kit (catalogue number 12727S). Cells were plated on substrates with stiffnesses of 2 kPa, 25 kPa, and 50 kPa and cultured overnight until reaching 40–50% confluence. The cells were then fixed with 4% formalin at room temperature for 15 min. After fixation, the cells were washed three times with PBS. Blocking was carried out with the kit's blocking agent for 1 h, followed by incubation with the primary antibody overnight at 4 °C. The next day, cells were washed thrice with PBS and incubated with fluorescence-conjugated secondary antibodies for 1 h at room temperature. After three additional washes, nuclear staining was performed using DAPI (Beyotime, BB-4133) for 5–30 min in the dark. The results were observed using a fluorescence microscope. Specific antibodies used are listed in Table S1.

### Tumour formation in nude mice

The Ethics Committee approved the animal experiments for Experimental Animals of Xiangya Hospital, Central South University (Changsha, China; approval number: 202103520), and adhered to internationally accepted principles for the care and use of experimental animals (NRC, 2011). Twenty-four 4-week-old male BALB/C nude mice were sourced from Beijing Vital River Laboratory Animal Technology Co., Ltd. After a one-week acclimation period, the mice were randomly divided into four groups, each consisting of six mice. For the tumour formation experiments, different cell treatments were injected subcutaneously into the left axillary region of the mice as follows: shControl-HCT116 + LVControl-CCD-18Co, shHSF4-HCT116 + LVControl-CCD-18Co, shControl-HCT116 + LVLOXL1-CCD-18Co, and shHSF4-HCT116 + LVLOXL1-CCD-18Co. Each mouse received a 100 µL injection containing a mixture of CCD-18Co cells (4 × 10^5^) and HCT116 cells (2 × 10^6^). Mice were weighed and monitored twice weekly. After 17 days, the experiment was concluded. Tumours were photographed, excised, and fixed in paraformaldehyde.

### Liver metastasis model construction

The same groups of 4-week-old male BALB/C nude mice were used for the liver metastasis experiment. Following a one-week acclimation period, the mice were randomly divided into four groups of six. The different treated cells were injected into the spleen of each mouse in the respective groups: shControl-HCT116 + LVControl-CCD-18Co, shHSF4-HCT116 + LVControl-CCD-18Co, shControl-HCT116 + LVLOXL1-CCD-18Co, and shHSF4-HCT116 + LVLOXL1-CCD-18Co. Each mouse received a mixture of CCD-18Co (4 × 10^5^) and HCT116 (2 × 10^6^) cells in 100 µL. Post-injection, the mice were monitored and weighed twice weekly. The experiment was concluded on day 19, and the liver and spleen were collected, weighed, and fixed in 4% paraformaldehyde.

### Statistical analysis

This study's statistical analysis and data visualisation were conducted using GraphPad Prism 9, R, IBM SPSS 22.0, ImageJ, Adobe Illustrator CC 2024 software and BioRender.com. Quantitative data are expressed as mean values with standard errors from at least three independent experiments. Comparisons between two data groups were performed using the student's t-test, while differences among multiple groups were evaluated using two-way ANOVA with Tukey's test. A significance level of *P* < 0.05 was considered statistically significant. Specific statistical methods and results are detailed in the figure legends. For example, in Gene Set Enrichment Analysis (GSEA), a false discovery rate (FDR) of 0.1 was applied to adjust for multiple comparisons. *P*-values were calculated based on two-sided statistical tests, with significance levels denoted as *P < 0.05, **P < 0.01, and ***P < 0.001. Results with *P* > 0.05 were considered not significant (ns).

## Results

### High tumour stiffness leads to poor prognosis in colorectal cancer

**Fig. 1:**
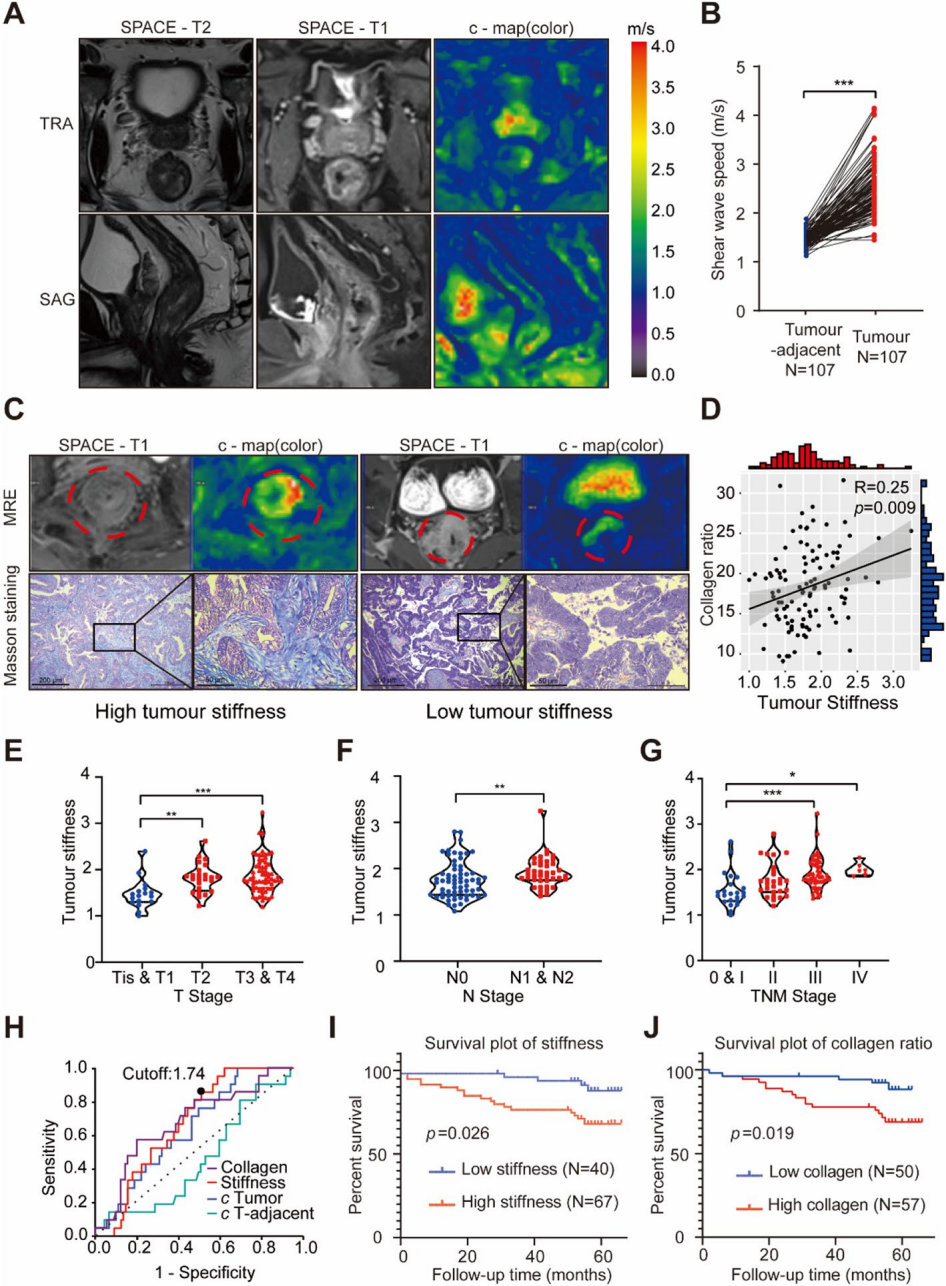
High Stiffness Leads to Poor Prognosis in Colorectal Cancer. **A** Representative MRI 3D SPACE T2w images, T1, MRE magnitude images, and c-maps (in colour) for CRC patients, presented in both transverse (TRA) and sagittal (SAG) views. **B** Line graph depicting shear wave velocity in tumour and adjacent normal tissues from 107 CRC patients. Tumour tissues exhibit significantly higher shear wave velocity than normal tissues (p-values determined using a matched T-test). **C** Representative MRE images and Masson staining results for High Tumour Stiffness and Low Tumour Stiffness. The upper red region denotes the tumour region of interest (ROI), while the lower panels display Masson staining images with scale bars of 200 μm and 50 μm, respectively. **D** Spearman correlation analysis between tumour stiffness and collagen ratio in 107 patients. The collagen ratio is quantified from Panel C (R = 0.25, *P* = 0.009). The grey area represents the 95% confidence interval. **E**–**G** Violin plots illustrate the relationships between tumour stiffness and T stage (**E**), N stage (**F**), and TNM stage (**G**). Elevated stages in all categories correlate with increased tumour stiffness. Statistical significance was determined using a two-tailed Student's t-test (*N* = 107 for all plots). H. Receiver Operating Characteristic (ROC) curve analysis was performed for collagen content, tumour stiffness, c-tumour, and c-tumour adjacent parameters based on patient death data. The Area Under the Curve (AUC) for tumour stiffness was 0.694, with the best cutoff value of 1.74. I-J. Kaplan–Meier plots comparing patients' overall survival (OS) based on tumour stiffness and collagen content. Patients with high stiffness and high collagen exhibit poorer survival rates than those with low stiffness and low collagen (evaluated using the log-rank test). For all panels: *p < 0.05, **p < 0.01, ***p < 0.001, ****p < 0.0001, and ns denotes not significant

Despite the growing evidence linking tumour stiffness with poor patient prognosis, the specific relationship between CRC stiffness and its biological behaviour and prognosis remains underexplored. This study included a cohort of 107 CRC patients with a mean follow-up period of 58 months. Each patient underwent MRE to measure the stiffness of the tumour and adjacent normal bowel tissue (Fig. 1A). Clinical and pathological data were collected, and the characteristics of the patients are summarised in Table 1. The results demonstrated that the shear wave velocity of the tumours was significantly higher than that of the adjacent normal bowel tissue (Fig. 1B).

**Table 1:** Patient Characteristics and Comparison between Low Collagen and High Collagen

Characteristics	All Patients (*N* = 107)	Low Collagen (*N* = 50)	High Collagen (*N* = 57)	*P*-value
Sex				
Male	62	28	34	0.85
Female	45	22	23	
Age (year)	56.92 ± 10.83	56.96 ± 12.19	56.88 ± 9.58	0.97
Height (m)	1.62 ± 0.07	1.61 ± 0.08	1.62 ± 0.07	0.44
Weight (kg)	59.76 ± 11.54	57.47 ± 11.32	61.76 ± 11.45	0.05
BMI (kg/m^2^)	22.74 ± 3.35	22.03 ± 3.30	23.35 ± 3.29	0.041*
Stiffness Characteristics				
Shear Wave Velocity of Normal Tissue (m/s)	1.28 ± 0.13	1.27 ± 0.12	1.29 ± 0.14	0.46
Shear Wave Velocity of Tumour Tissue (m/s)	2.30 ± 0.56	2.18 ± 0.46	2.41 ± 0.61	0.028*
Ratio of Tumour/Normal	1.80 ± 0.39	1.72 ± 0.31	1.88 ± 0.43	0.031*
MRI Characteristics				
mT				
mT1	2	0	2	0.041*
mT2	23	13	10	
mT3	66	34	32	
mT4	16	3	13	
mN				
mN0	49	24	25	0.08
mN1	38	21	17	
mN2	20	5	15	
mM				
mM0	100	48	52	0.45
mM1	7	2	5	
Circumferential Resection Margin				
Positive	15	5	10	0.40
Negative	92	45	47	
Extramural Vascular Invasion;				
Positive	28	8	20	0.029*
Negative	79	42	37	
Tumour Growth Pattern				
Intramural	32	21	11	0.013*
Luminal	64	27	37	
Both	11	2	9	
Tumour Length (mm)	43.53 ± 16.72	40.62 ± 14.97	46.09 ± 17.86	0.09
Tumour Thickness (mm)	15.39 ± 7.89	13.34 ± 4.98	17.19 ± 9.44	0.009**
MRI Cancer Nodule				
Positive	10	3	7	0.33
Negative	97	47	50	
Hyperintensity on T2w images				
Positive	14	10	4	0.08
Negative	93	40	53	
Vascular Edema, Necrosis or Mucous Degeneration				
Positive	20	9	11	1.00
Negative	87	41	46	
Hemorrhage, High T1				
Positive	2	2	0	0.21
Negative	105	48	57	
Number of Mesorectal Lymph Nodes				
0	48	24	24	0.11
1	14	9	5	
2	22	11	11	
> = 3	23	6	17	
Histopathologic features				
pT or ypT				
Tis	7	0	7	0.06
T1	10	7	3	
T2	24	10	14	
T3	63	31	32	
T4	3	2	1	
pN or ypN				
N0	68	30	38	0.67
N1	32	17	15	
N2	7	3	4	
TNM stage or ypTNM stage				
0	7	0	7	0.001**
I	16	11	5	
II	27	19	8	
III	51	18	33	
IV	6	2	4	
Cancer Nodule				
Positive	12	5	7	0.76
Negative	95	45	50	
Perineural Invasion				
Positive	15	6	9	0.78
Negative	92	44	48	
Vascular Invasion				
Positive	18	9	9	0.80
Negative	89	41	48	
Histologic Grade				
Low	13	6	7	0.07
Moderate	82	42	40	
High	2	1	1	
Unassessable or Missing	10	1	9	
Mismatch Repair Status				
Deficient Mismatch Repair	9	6	3	0.20
Proficient Mismatch Repair	76	37	39	
Unassessable or Missing	22	7	15	
Progression				
Locally advanced rectal cancer	84	39	45	1.00
Non-locally advanced rectal cancer	23	11	12	
Survival Status				
Alive	86	50	57	0.027*
Deceased	21	5	16	
Average Survival Time (months)	58.63 ± 1.61	60.52 ± 1.34	54.63 ± 2.64	0.019*

Notes: This table comprehensively presents the demographic, clinical, and histopathologic characteristics of all patients (*N* = 107), stratified by collagen levels into the Low Collagen (*N* = 50) and High Collagen (*N* = 57) groups. Categorical variables were compared using Chi-square tests, while continuous variables were analysed with student's t-tests (or suitable non-parametric tests, where applicable, when normality assumptions were violated). Abbreviations used throughout the table include MRI (Magnetic Resonance Imaging) for imaging traits, BMI (Body Mass Index) for anthropometric measurements, and pT, pN, and TNM for pathological staging. Abbreviations mT, mN, and mM refer to MRI-based tumour, node, and metastasis staging. In this study, a *P*-value less than 0.05 was considered statistically significant. An asterisk (*) signifies a statistically significant difference (*p* < 0.05), while a double asterisk (**) indicates a higher level of significance (e.g., *p* < 0.01)

Given the strong correlation between tumour stiffness and collagen content, we further investigated this relationship by performing Masson's staining on samples from each patient and analysing the correlation between MRE results and collagen content (Fig. 1C). Tumour stiffness was quantified as the ratio of tumour stiffness to normal tissue stiffness to minimise the influence of each patient's normal tissue stiffness. The results of Masson staining indicated a positive correlation between MRE findings and tumour collagen ratio (Fig. 1D).

Moreover, our study revealed a close association between tumour stiffness and tumour malignancy, as evidenced by significant correlations with T, N, and TNM stages (Fig. 1E-G). Through ROC curve analysis, combined with patient mortality data, we identified optimal cutoff values for tumour stiffness and collagen ratio (Fig. 1H). Patients with a stiffness ratio greater than 1.74 were classified into the high-stiffness group, while those with a ratio of 1.74 or less were placed in the low-stiffness group. Kaplan–Meier (K-M) survival analysis showed that the mean survival time in the high-stiffness group was 54.38 ± 2.64 months, compared to 64.05 ± 1.00 months in the low-stiffness group (*P* = 0.007) (Fig. 1I). Similarly, patients with a collagen ratio exceeding 17.6% were classified into the high-collagen group, while those with 17.6% or less were classified into the low-collagen group. Specifically, the mean survival time was 54.63 ± 2.64 months in the high-collagen group and 60.52 ± 1.34 months in the low-collagen group (*P* = 0.019) though K-M survival analysis (Fig. 1J). These findings suggest that tumour stiffness and collagen content are closely related to CRC prognosis and may be effective indicators of cancer progression and patient outcomes.

### The effects of different extracellular matrix stiffness on colorectal cancer morphology and gene expression

**Fig. 2:**
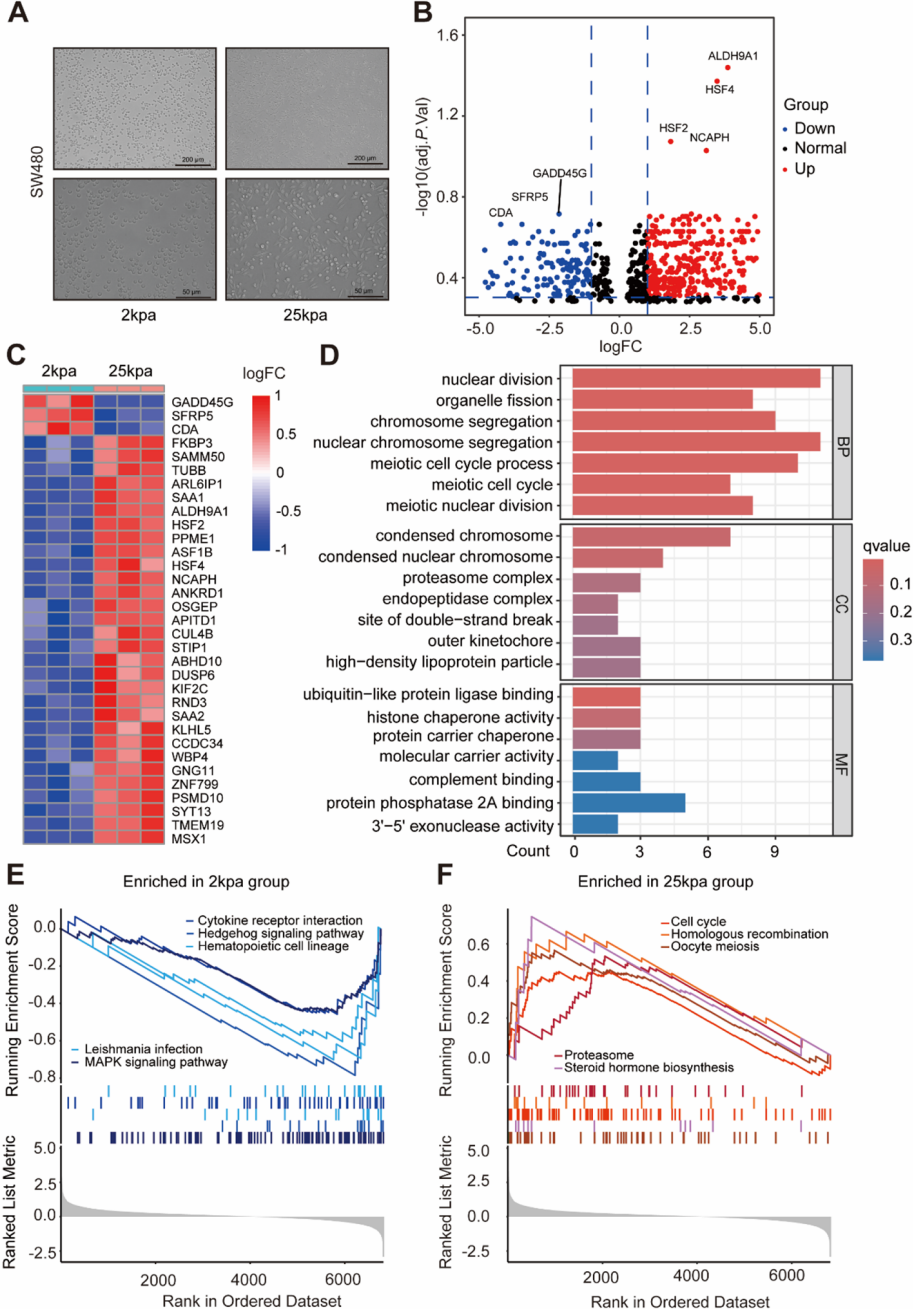
Extracellular Matrix Stiffness Significantly Affects Gene Expression in CRC Cell Lines. **A** Significant morphological differences were observed under an optical microscope After culturing SW480 cell lines for 72 h in media with 2 kPa and 25 kPa stiffness levels. In soft matrices, cells exhibited a spherical shape, whereas in stiff matrices, cells became polygonal with pseudopodia formation. Scale bars: 200 μm (upper panel), 50 μm (lower panel). **B** The volcano plot displays the top 1000 differentially expressed genes, with red indicating genes upregulated in high-stiffness matrices and blue indicating genes upregulated in low-stiffness matrices, highlighting significant gene expression differences. **C** The heatmap illustrates the 33 most differentially expressed genes. Among these, three genes are downregulated in low-stiffness conditions, while thirty genes are significantly upregulated in high-stiffness conditions. **D** GO functional annotation analysis reveals the impact of extracellular matrix stiffness on Biological Processes (BP), Cellular Components (CC), and Molecular Functions (MF). **E** ssGSEA analysis shows five signalling pathways significantly downregulated under 2 kPa extracellular matrix conditions. **F** ssGSEA analysis shows that five signalling pathways are significantly upregulated under 25 kPa extracellular matrix conditions. For all panels: *p < 0.05, **p < 0.01, ***p < 0.001, ****p < 0.0001, and ns denotes not significant

To further elucidate the molecular mechanisms by which ECM stiffness affects the biological behaviour of CRC, we cultured the SW480 cell line on matrices with stiffness levels of 2 kPa and 25 kPa, respectively. Significant changes in cell morphology were observed under different stiffness conditions (Fig. 2A). Transcriptome deep sequencing analysis revealed 1,759 genes with significantly altered expression (Fig. 2B), with the top 33 differentially expressed genes including GADD45G, SFRP5, and CDA. (Fig. 2C). Functional annotation indicated that high stiffness notably impacted biological processes, particularly nuclear division and organelle fission (Fig. 2D). Single-sample Gene Set Enrichment Analysis (ssGSEA) showed that high ECM stiffness significantly affected various cellular functions, such as activating the cell cycle and homologous recombination, while low stiffness inhibited pathways like cytokine-cytokine receptor interaction and hedgehog signalling pathway(Fig. 2E, F). The deep sequencing and processed data have been uploaded to the NCBI Sequence Read Archive (SRA) database under PRJNA522032 and the Gene Expression Omnibus (GEO) database under accession number GSE273846. These results indicate that the mechanical properties of the ECM play a crucial role in regulating cell behaviour, especially within the tumour microenvironment. High ECM stiffness may promote tumour progression by enhancing cell proliferation and DNA repair mechanisms, leading to a more aggressive cancer phenotype.

### High expression of HSF4 in CRC is significantly associated with tumour stiffness and predicts poor prognosis

**Fig. 3:**
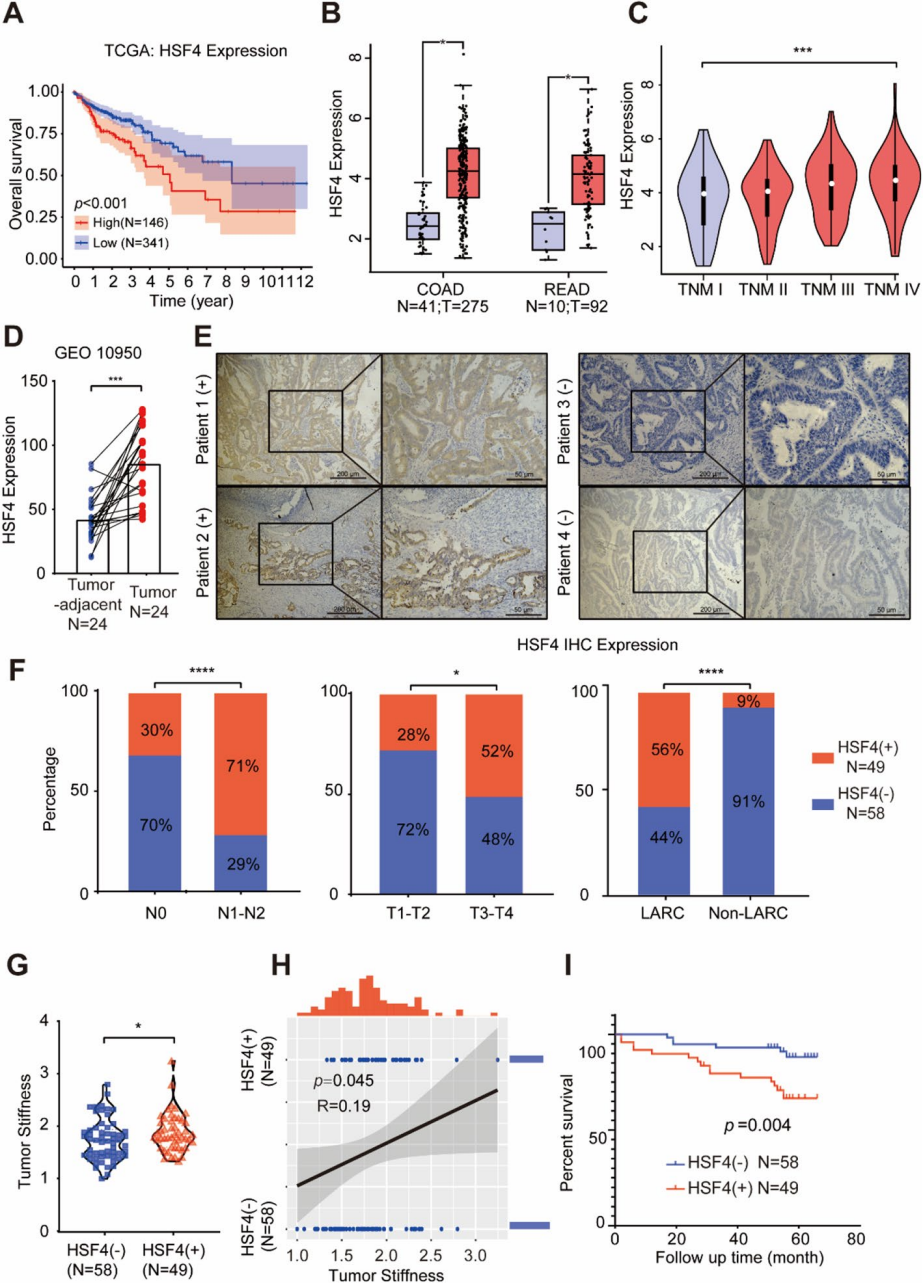
HSF4 in CRC is Closely Linked to Tumour Stiffness and Indicates Poor Prognosis. **A** Kaplan–Meier plot comparing the overall survival (OS) of TCGA CRC patients with high and low HSF4 expression (*N* = 487). The log-rank test was used for comparison. **B** Comparison of HSF4 expression in the TCGA COAD (colon adenocarcinoma) (*N* = 41, T = 275) and READ (rectum adenocarcinoma) (*N* = 10, T = 92) datasets. N represents normal colorectal samples, and T represents tumour samples. Student's t-test was used for comparison. The log2(TPM + 1) transformation was applied for logarithmic presentation. **C** Assessment of the relationship between log2(TPM + 1) transformed HSF4 expression and CRC TNM staging using the Gepia2 database. An F-test was conducted, with F value = 5.36 and Pr(> F) = 0.00128. **D** HSF4 expression in tumour and adjacent normal tissues from 24 CRC patients. A paired samples t-test was used for comparison. **E** Representative IHC images of HSF4 expression in tumour tissues from 4 patients in our cohort. Patients 1, 2, and 3 showed positive HSF4 expression, while Patient 4 showed negative expression. Scale bars are 200 μm and 50 μm, respectively. **F** Association between HSF4 expression and N stage, T stage, and LARC (locally advanced rectal cancer) and non-LARC in 107 patients from our cohort. A Chi-square test was used for comparison. **G** Relationship between HSF4 IHC expression and tumour stiffness in 107 patients. A t-test was used for comparison. **H** Correlation between HSF4 IHC results and tumour stiffness. Spearman correlation test was used for analysis.** I** Kaplan–Meier plot comparing the overall survival (OS) of CRC patients with high and low HSF4 expression in our cohort (*N* = 107). The log-rank test was used for comparison. For all panels: *p < 0.05, **p < 0.01, ***p < 0.001, ****p < 0.0001, and ns denotes not significant

We conducted a detailed analysis of the sequencing results for the 33 most significantly differentially expressed genes to identify the key genes that may impact CRC prognosis and those regulated by tumour stiffness. Our study revealed a strong association between the high expression of Heat Shock Transcription Factor 4 (HSF4) and increased tumour stiffness, indicating a poor prognosis. Kaplan–Meier survival analysis using the TCGA database showed that patients with high HSF4 expression had significantly worse outcomes (Fig. 3A). Additionally, HSF4 expression was significantly higher in tumour tissues compared to normal tissues in both colon and rectal cancers (Fig. 3B). Moreover, HSF4 expression increased with advancing TNM stage (Fig. 3C).

To validate these findings across different datasets, we also analysed the GEO10950 dataset from 24 CRC patients and confirmed that HSF4 expression was elevated in tumour tissues compared to adjacent normal tissues. To further elucidate the clinical significance of HSF4 expression in CRC, we performed immunohistochemistry (IHC) for HSF4 on samples from 107 patients in our study cohort, with representative images shown in Fig. 3E. In this cohort, we observed higher HSF4 protein expression in metastatic lymph nodes, with its positivity rate increasing with more advanced T stages. Moreover, the positivity rate was significantly higher in locally advanced rectal cancer (LARC) than in non-LARC cases (Fig. 3F).

We also investigated the correlation between HSF4 IHC positivity and tumour stiffness. The results demonstrated that tumours from HSF4-positive patients exhibited higher stiffness (Fig. 3G), with a significant correlation between these two factors (Fig. 3H). K-M analysis of our cohort further showed that HSF4-positive patients had a poorer prognosis, with a mean survival time of 54.04 ± 2.90 months compared to 62.43 ± 1.51 months for HSF4-negative patients (*P* < 0.001). Our cohort also revealed that HSF4 positivity was not associated with the tumour collagen ratio (Figure S1). These findings suggest that HSF4 is closely linked to increased tumour stiffness, and its high expression predicts poor prognosis in CRC.

### HSF4 mediates CRC Invasion and metastasis by responding to extracellular matrix stiffness

**Fig. 4:**
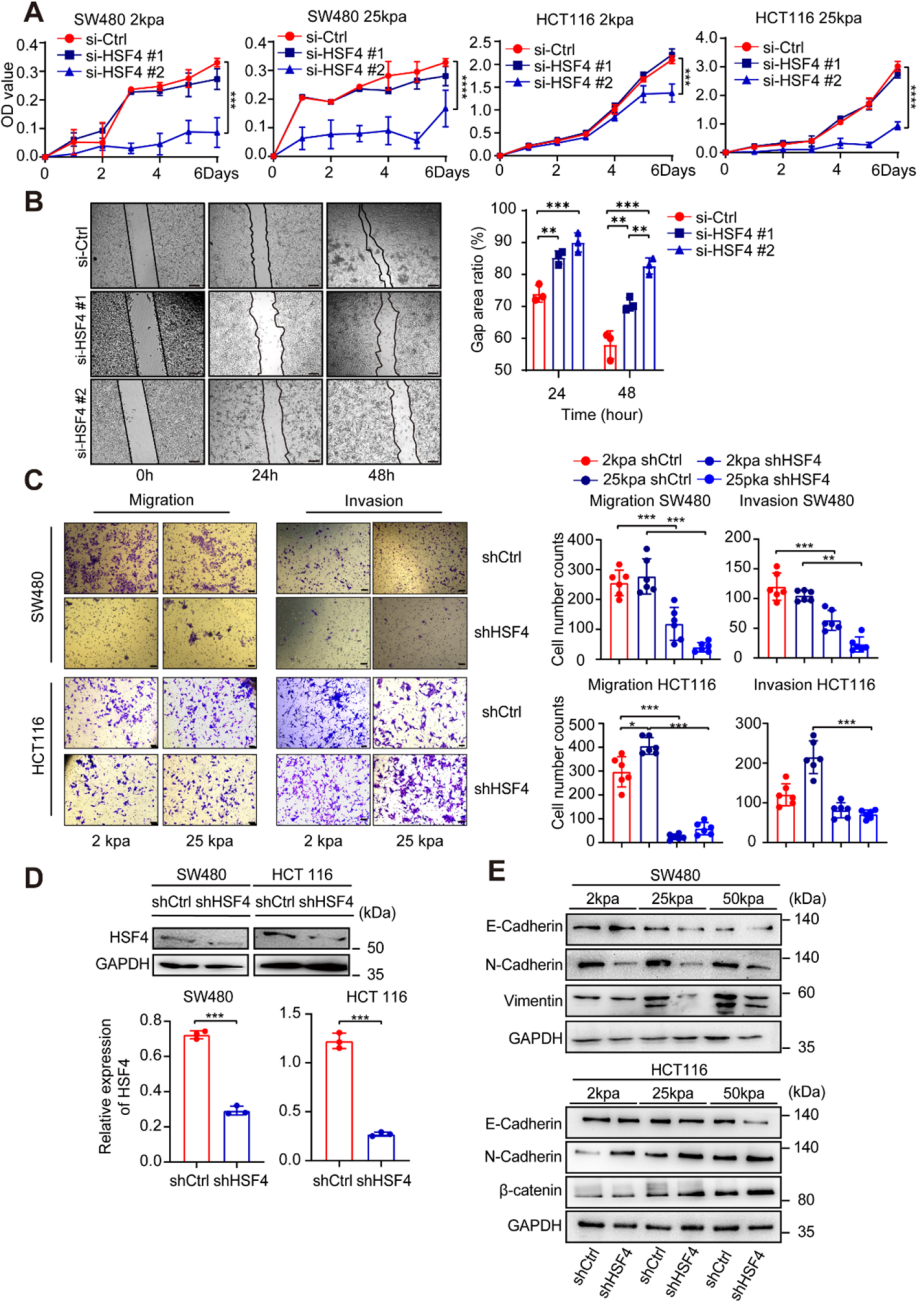
HSF4 Knockout Affects CRC Cell Behavior and EMT Marker Expression in Response to Matrix Stiffness. **A** CCK-8 assay curves for SW480 and HCT116 cell lines cultured on 2 kPa and 25 kPa matrices. si-Ctrl represents the control group, while si-HSF4 #1 and si-HSF4 #2 are the experimental groups. Experiments were repeated three times, and data are presented as mean ± SD. *P*-values were determined using a two-tailed Student's t-test. **B** Representative images and quantified data from wound healing assays. si-Ctrl represents the control group, while si-HSF4 #1 and si-HSF4 #2 are the experimental groups. Data are presented as mean ± SD. *P*-values were determined using a two-tailed Student's t-test. Scale bars: 200 μm. **C** Representative images and quantified data from Migration and Invasion assays of SW480 and HCT116 cell lines on 2 kPa and 25 kPa matrices. Experiments were repeated six times, presenting data as mean ± SD. *P*-values were determined using a two-tailed Student's t-test. Scale bars: 200 μm. **D** Western blot and quantified data results showing the establishment of HSF4 knockout cell lines in SW480 and HCT116. Data are presented as mean ± SD. *P*-values were determined using a two-tailed Student's t-test. Scale bars: 200 μm. **E** Western blot results for E-Cadherin, N-Cadherin, and Vimentin (SW480), and β-catenin (HCT116) in HSF4 knockout cell lines cultured on 2 kPa, 25 kPa, and 50 kPa matrices. For all panels: *p < 0.05, **p < 0.01, ***p < 0.001, ****p < 0.0001, and ns denotes not significant

The correlation between HSF4 expression and CRC tumour stiffness and its association with poor prognosis prompted us to investigate further. To explore the functional role of HSF4, we established HSF4 knockout models in SW480 and HCT116 cell lines and examined their behaviour under different stiffness conditions. We found that HSF4 knockout significantly reduced cell proliferation, irrespective of substrate stiffness (Fig. 4A). The wound healing assay demonstrated that the migratory capacity of tumour cells was markedly impaired following HSF4 knockout (Fig. 4B). Migration and invasion assays conducted on extracellular matrices of varying stiffness revealed that HSF4 enhances cell invasion and migration in both CRC cell lines, with these effects becoming more pronounced as stiffness increases (Fig. 4C).

We also employed Western blot analysis to evaluate the efficiency of HSF4 knockdown using siHSF4 #1 and siHSF4 #2 (Figure S2). We ultimately selected siHSF4 #2 based on efficiency and stability to establish a stable HSF4 knockout cell line (Fig. 4D). We then analysed the protein expression of EMT-related proteins under different stiffness conditions (2, 25, and 50 kPa). The results showed that as stiffness increased, E-cadherin expression decreased while N-cadherin expression increased. The result suggests that stiffness induces EMT in CRC, promoting invasion and metastasis (Fig. 4E, Figure S3). Knockdown of HSF4 significantly inhibits the expression of EMT marker proteins (N-Cadherin and Vimentin) while increasing E-Cadherin expression. However, SW480 cells exhibit a more stable response to HSF4 knockdown, with minimal influence from substrate stiffness. In contrast, the response of HCT116 cells to HSF4 knockdown is more significantly modulated by variations in substrate stiffness. Moreover, HSF4 expression and increased stiffness promoted the nuclear translocation of β-catenin (Figure S4), and overexpression of HSF4 promotes migration and invasion abilities in SW480 and HCT116 (Figure S5). These findings indicate that HSF4 responds to extracellular matrix stiffness by regulating EMT-related signalling pathways, thereby promoting CRC invasion and metastasis.

In conclusion, these results underscore the critical role of HSF4 in sensing extracellular matrix stiffness and promoting CRC invasion and metastasis via EMT-related signalling pathways.

### HSF4 promotes CRC growth independently of collagen in nude mouse

**Fig. 5:**
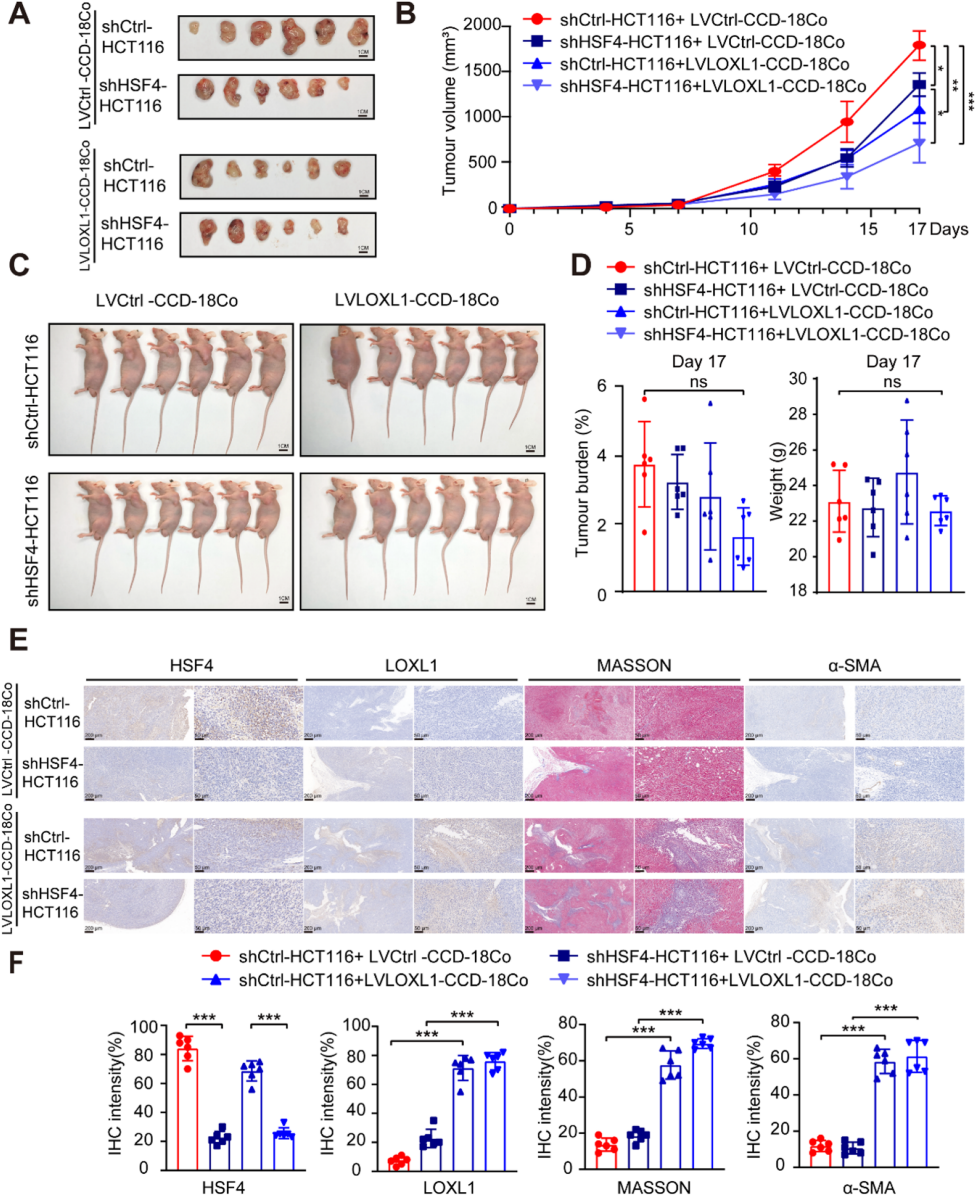
Tumour-Promoting Effect of HSF4 in vivo. **A** Images of excised tumours from each group of six mice (*n* = 6). Scale bars: 1 cm. **B** Tumour growth curves for each group of mice (*n* = 6). Data are presented as mean ± SD. *P*-values were determined using a two-tailed Student's t-test. **C** Images of mice at the time of sacrifice, with each group comprising six mice (*n* = 6). Scale bars: 1 cm. **D** Tumour burden (%) and weight (g) at day 17 were calculated as tumour weight/mouse body weight for each group of mice (*n* = 6). Data are presented as mean ± SD. *P*-values were determined using a two-tailed Student's t-test. **E** Representative HSF4, LOXL1, MASSON, and α-SMA staining images in tumour tissues from (A). Scale bar, 200 μm left and 50 μm right. **F** Quantification of (**E**). Data are presented as mean ± SEM. *P*-values were determined using a two-tailed Student's t-test. For all panels: *p < 0.05, **p < 0.01, ***p < 0.001, ****p < 0.0001, and ns denotes not significant

Cancer-associated fibroblasts (CAFs) are crucial in the extracellular matrix [29, 30]. To investigate how HSF4 promotes tumour growth in this context, we knocked out HSF4 in HCT116 cells and co-seeded with fibroblasts (CCD-18Co), followed by in vivo tumourigenesis experiments in nude mice. Lysyl Oxidase Like 1 (LOXL1), a member of the lysyl oxidase family, catalyses the oxidation of lysine residues in collagen and elastin, promoting cross-linking in the ECM [31–34]. We overexpressed LOXL1 in CCD-18Co cells (Figure S6) to assess its correlation with HSF4. Results showed that HSF4 knockout reduced tumour size, and unexpectedly, the combination of HSF4 knockout and LOXL1 overexpression further reduced tumour proliferation (Fig. 5A, B). Additionally, the in vivo tumourigenesis experiment did not significantly impact the mice's tumour burden or weight (Fig. 5 C, D).

To further assess the ECM environment of the tumours, we performed immunohistochemical staining to evaluate the expression of HSF4, LOXL1, MASSON, and α-SMA. The IHC results confirmed the establishment a stable cell line(Fig. 5E, F). MASSON staining indicated that collagen content increased with LOXL1 expression. Furthermore, high levels of LOXL1 promoted α-SMA expression (Fig. 5 E, F). These findings suggest that HSF4 knockout in HCT116 cells seeded with CCD-18Co fibroblasts reduces tumour growth, while LOXL1 overexpression amplifies this effect, leading to even smaller tumours. This unexpected result indicates that high LOXL1 levels promote ECM collagen and fibroblast differentiation into myofibroblasts, characterised by increased α-SMA expression. Although LOXL1-induced ECM collagen and myofibroblast content increased, tumour proliferation was inhibited. These results indicate that HSF4 promotes tumour growth independently of ECM collagen content and highlights the complex tumour-stroma interactions within ECM dynamics.

### HSF4 Promotes distant metastasis of CRC Independently of collagen

**Fig. 6:**
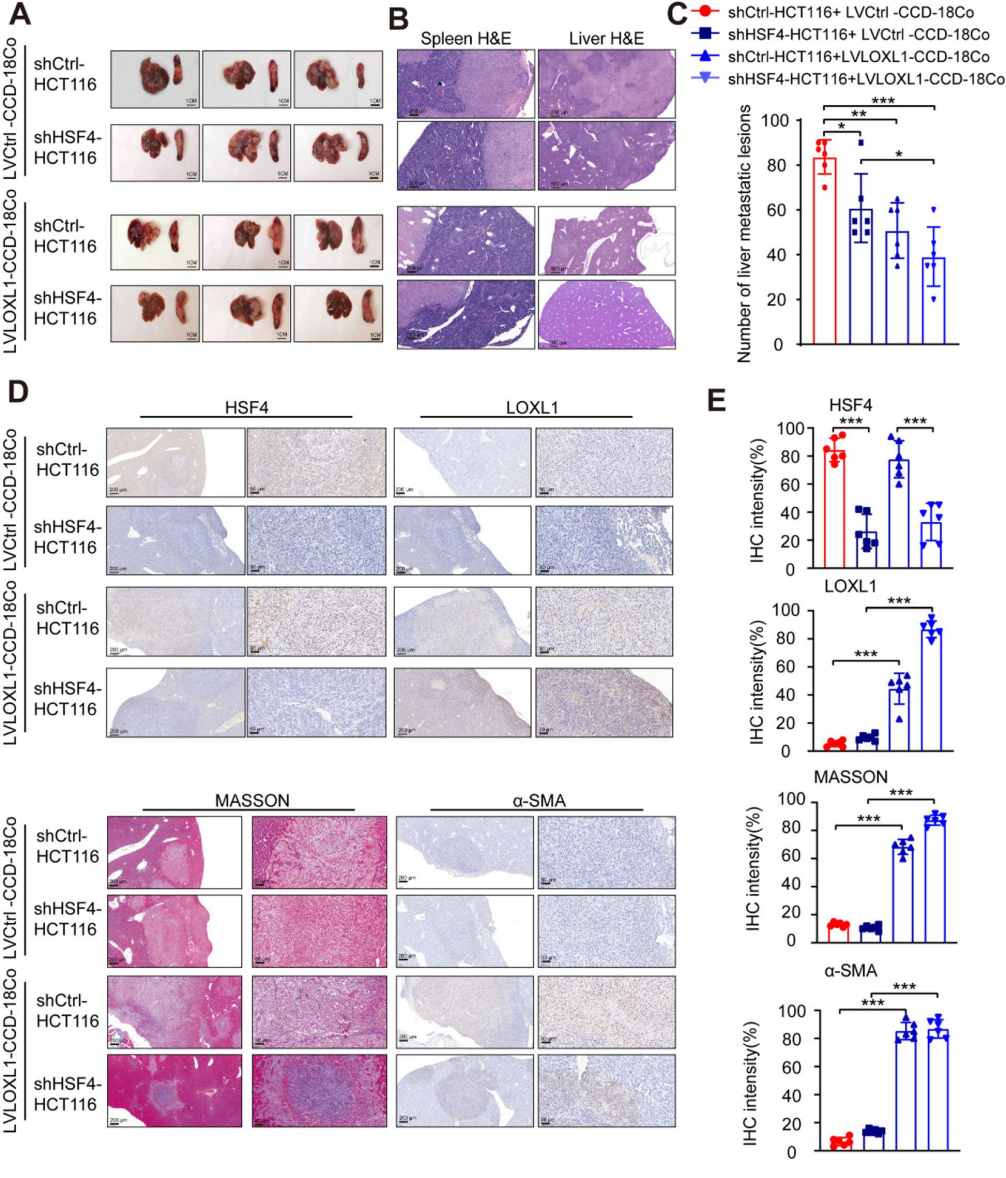
HSF4 Promotes CRC Distant Metastasis in a Mouse Liver Metastasis Model. **A** Representative images of excised spleens and livers from each group of mice (*n* = 6 per group). Scale bar: 1cm. **B** Representative H&E-stained images of metastatic lesions in the spleen and liver. Scale bars: left, 200 μm; right, 500 μm. **C** Quantification of liver metastatic lesions shown in (B). Data are presented as mean ± SD, with p values determined using a two-tailed Student's t-test. **D** Representative HSF4, LOXL1, MASSON, and α-SMA staining images in liver metastatic lesions from (A). Scale bars: left, 200 μm; right, 50 μm. **E** Quantification of staining in (D). Data are presented as mean ± SEM, with p values determined using a two-tailed Student's t-test. For all panels: *p < 0.05, **p < 0.01, ***p < 0.001, ****p < 0.0001, and ns denotes not significant

Given that HSF4 promotes tumour growth independently of collagen, we further explored the role of HSF4 and CAFs in the distant metastasis of CRC. We knocked out HSF4 in HCT116 cells and cocultured them with LOXL1-overexpressing CCD-18Co cells, followed by liver metastasis experiments in nude mice. The results showed that HSF4 knockout significantly reduced the number of liver metastases and, combined with LOXL1 overexpression, further decreased the number of metastatic tumours in the liver. Additionally, reduced tumour invasion ability was observed in the spleen (Fig. 6 A, B, C).

**Fig. 7:**
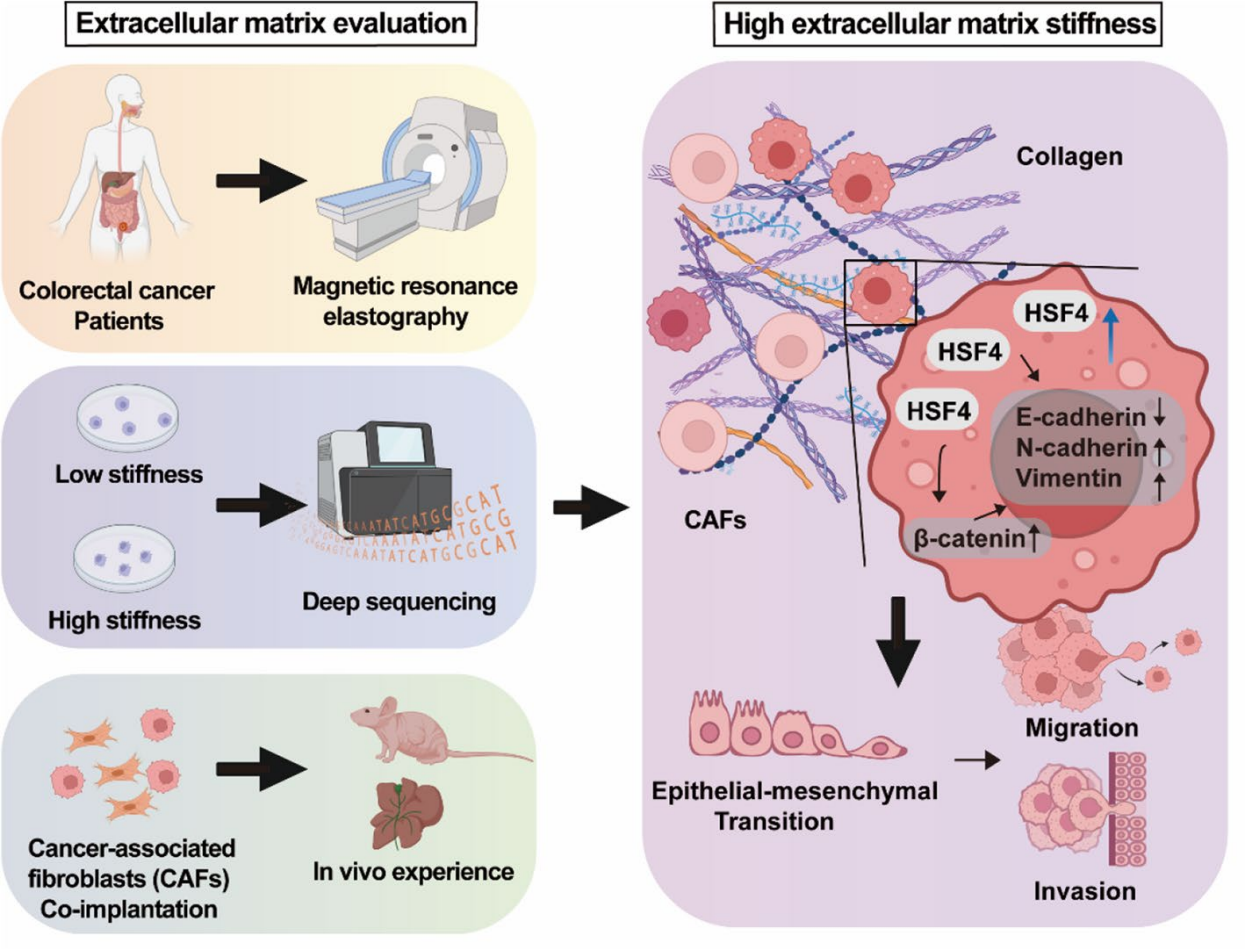
Flowchart and schematic diagram of research processes and mechanisms

To further evaluate the impact of the ECM environment on CRC liver metastasis, we performed immunohistochemical staining on liver metastases to assess the expression of HSF4, LOXL1, MASSON, and α-SMA. The results showed that HSF4 expression decreased following knockout, while LOXL1 expression was consistent with overexpression (Fig. 6 D, E). MASSON staining indicated that collagen content increased with LOXL1 expression, and α-SMA expression was positively correlated with LOXL1. High levels of LOXL1 promoted α-SMA expression. These findings suggest that HSF4 promotes distant metastasis in CRC, and the combined effect of HSF4 knockout and LOXL1 overexpression further reduces the invasive ability of distant tumours. Similar to the in vivo tumourigenesis results, tumour proliferation was inhibited despite the increase in ECM collagen and myofibroblast content induced by LOXL1. These results indicate that HSF4 promotes tumour metastasis independently of ECM collagen content, highlighting the complex balance of tumour-stroma interactions within ECM dynamics. Finally, the research process and the role of HSF4 in extracellular matrix stiffness are illustrated in Fig. 7.

## Discussion

This study comprehensively explores the role of tumour stiffness and its molecular basis in the progression and prognosis of CRC. By utilising MRE to measure tumour stiffness and Masson staining to assess collagen content, we demonstrated that higher stiffness, reflected in increased shear wave velocity and collagen content, is significantly associated with advanced TNM staging and lower patient survival rates. Furthermore, we investigated the impact of ECM stiffness on CRC cell behaviour through in vitro culture experiments, revealing that high ECM stiffness promotes cell proliferation, migration, and invasion and alters gene expression profiles, particularly by upregulating HSF4. High HSF4 expression correlates closely with tumour stiffness and predicts poor prognosis in CRC patients. Functional studies indicated that HSF4 responds to ECM stiffness and promotes CRC invasion and metastasis through EMT-related signalling pathways. Importantly, HSF4 promotes CRC growth and metastasis independently of collagen content, highlighting the complex interactions between tumour cells and their microenvironment. Our findings underscore the critical role of tumour stiffness and ECM mechanics in CRC progression and suggest that HSF4 is a potential therapeutic target.

Current research shows a strong correlation between tumour stiffness and prognosis across various solid tumours, particularly in breast [35], prostate [36], liver cancer [37] and CRC [38]. Increased tumour stiffness often indicates a poorer prognosis. In breast cancer studies, tumour stiffness assessed via palpation or ultrasound elastography is confirmed as an independent prognostic factor, closely related to tumour invasiveness, lymph node metastasis, and distant metastasis risk. Similarly, studies in prostate cancer have used MRI-guided elastography to reveal significant associations between tumour stiffness, Gleason scores, and disease recurrence, emphasising the pivotal role of physical properties of the tumour microenvironment in cancer progression [39]. Various methods are employed to measure tumour stiffness, including optical coherence tomography (OCT) [40], atomic force microscopy (AFM) [41], acoustic radiation force impulse imaging (ARFI) [42], and shear wave elastography (SWE) [43]. Despite its limited penetration depth, OCT shows potential in assessing superficial tumour stiffness due to its high resolution [40]. AFM can directly measure nanoscale mechanical properties of surfaces, and it is widely used in basic research despite its complexity [41]. ARFI and SWE, as extensions of ultrasound technology, evaluate tissue elasticity using acoustic radiation force and shear wave speed, offering non-invasive, real-time, and reproducible measurements widely applied in clinical diagnostics [42, 43]. This study utilised MRE technology to non-invasively and accurately measure tumour stiffness in CRC patients, providing comprehensive information on CRC stiffness and distribution, supporting early warning and personalised treatment planning.

By establishing a soft and stiff matrix culture model combined with deep sequencing, we systematically investigated the impact of ECM stiffness on the biological characteristics of CRC cells. This molecular-level study revealed the complex regulatory mechanisms by which ECM stiffness influences CRC cell behaviour. Our research identified differential expression of HSF4 and uncovered significant expression differences in several other genes, including MSX1 and DUSP6, under varying stiffness conditions. These findings enhance existing research and further refine the gene expression profiles associated with ECM stiffness. Previous studies have shown that regulating DUSP6 expression is closely linked to cell signalling, particularly the ERK/MAPK pathway, and may modulate the malignant phenotype of cancer cells by affecting cell proliferation and migration [44]. The sustained expression of MSX1 in regenerative cells is closely related to tissue regeneration and differentiation, with its differential expression under different ECM stiffness potentially reflecting its crucial role in regulating cell differentiation and regenerative potential [45]. Transcriptome sequencing data further revealed significant pathway alterations under conditions of increased stiffness, particularly the activation of the cell cycle and homologous recombination pathways, providing strong support for the role of ECM stiffness as a critical regulatory factor in the tumour microenvironment [46]. These findings deepen our understanding of how ECM stiffness regulates CRC cell behaviour and offer new molecular targets and strategies for future CRC therapies.

HSF4 plays a critical role in both the normal formation of the lens and the progression of tumours [47, 48]. It regulates the differentiation of lens fibre cells by activating p53 and its downstream regulators [49]. Additionally, high expression levels of HSF4 have been shown to promote the progression of pancreatic cancer and CRC [47]. For instance, the interaction between FGD3 and HSF4 can inhibit the expression of p65, thereby suppressing pancreatic cancer progression [50]. Our study further reveals that HSF4 can respond to matrix stiffness to promote CRC invasion and metastasis. However, an unexpected finding in our mouse model showed that CRC cell proliferation and invasiveness significantly decreased when cocultured with CAFs that promote the expression of LOXL1. Our results suggest that the mechanisms by which HSF4 influences CRC progression may be more complex than initially anticipated. Our study further advances the understanding of tumour stiffness mechanisms, revealing that the role of HSF4 is not entirely dependent on collagen, suggesting that stiffness may be influenced by additional factors that even interreact with immune cells. Concurrently, recent research targeting the LOXL family in anti-fibrosis treatments, particularly studies on Simtuzumab targeting LOXL2, has demonstrated that these therapies have not shown significant efficacy in combating fibrosis [51]. Tumour-associated immune cells, such as Osr2, which promotes CD8 + T cell exhaustion, also play a crucial role in tumour stiffness and immune evasion [52]. As a key member of the heat shock transcription factor family, HSF4 may indirectly affect the characteristics of the tumour microenvironment by regulating downstream target genes, thereby enhancing tumour stiffness and malignancy without directly increasing collagen content. This finding not only deepens our understanding of HSF4's role in CRC progression but also broadens our perspective on the mechanisms of tumour stiffness. It underscores the complexity and dynamism of the tumour microenvironment and suggests that future research should comprehensively consider all components and interactions to uncover deeper molecular mechanisms and develop more effective targeted therapies.

Despite significant progress in understanding the relationship between CRC stiffness, our study has limitations. Firstly, although our mean follow-up period was 58 months, the relatively limited sample size provides valuable insight for prognostic analysis and might not entirely reflect treatment benefits due to extended patient survival with advancements in medical technology. Future research should consider Progression-Free Survival (PFS) as a supplementary or alternative indicator to more accurately depict disease control during treatment [53]. Moreover, our sample collection from a single medical centre might limit the generalizability of the results. Conducting larger-scale and multi-centre clinical trials is essential to enhance the reliability and generalizability of conclusions. Secondly, although we have identified the critical role of HSF4 in tumour stiffness sensing and disease progression in CRC, the underlying molecular mechanisms and signalling pathways remain incompletely elucidated. In particular, while we have observed key molecular changes associated with EMT, the precise mechanisms by which HSF4 regulates EMT through pathways such as the TGF-β, Wnt/β-catenin, and Notch signalling pathways require further in-depth investigation [54–57]. Integrating MRE with molecular markers to develop more precise early diagnostic tools and personalised treatment plans is a crucial direction for future research. Finally, the in vivo models used in our study were immunodeficient mice, which effectively minimised interference from the immune system. However, the interaction between tumour stiffness and the immune system remains an area that warrants more detailed exploration. Future studies using immunocompetent mouse models are necessary to uncover the role of HSF4 within the immune microenvironment comprehensively. Constructing multilayer regulatory network models to comprehensively reveal the role of tumour stiffness in CRC development and progression will open new avenues for CRC prevention and treatment.

## Conclusion

This study comprehensively explores the role of tumour stiffness in CRC progression and prognosis. Using MRE and Masson staining, we demonstrated that high stiffness, reflected in increased shear wave velocity and collagen content, is significantly associated with poorer patient prognosis. Transcriptome analysis identified HSF4 as a key regulator of stiffness-mediated CRC behaviour. HSF4 expression correlates closely with tumour stiffness and predicts poor prognosis in CRC patients, promoting CRC invasion and metastasis through EMT-related signalling pathways independently of collagen content. These findings highlight the importance of the mechanical properties of the tumour microenvironment in CRC progression and suggest that HSF4 is a potential therapeutic target.

## Supplementary Information

Additional file 1.

Additional file 2.

## Data Availability

The article and Supplementary Material includes the original contributions presented in this study. Further inquiries can be directed to the corresponding author. The sequencing data have been uploaded to the NCBI BioProject database under accession number PRJNA522032 (access link: https://www.ncbi.nlm.nih.gov/bioproject/PRJNA522032 ). The processed data have been uploaded to the Gene Expression Omnibus (GEO) database under accession number GSE273846 (access link: https://www.ncbi.nlm.nih.gov/geo/query/acc.cgi?acc=GSE273846 ).

## Abbreviations

α-SMA: Alpha-Smooth Muscle Actin
CAFs: Cancer-Associated Fibroblasts
CCD-18Co: Colon Fibroblast cell line
CCK-8: Cell Counting Kit-8
CRC: Colorectal Cancer
ECL: Enhanced Chemiluminescence
ECM: Extracellular Matrix
EMT: Epithelial-Mesenchymal Transition
FDA: Food and Drug Administration
FPKM: Fragments Per Kilobase of exon model per Million mapped reads
GEO: Gene Expression Omnibus
GO: Gene Ontology
GSEA: Gene Set Enrichment Analysis
HSF4: Heat Shock Transcriptional Factor 4
IHC: Immunohistochemistry
LOXL1: Lysyl Oxidase Like 1
MRE: Magnetic Resonance Elastography
MRI: Magnetic Resonance Imaging
OS: Overall Survival
PFS: Progression-Free Survival
PVDF: Polyvinylidene Fluoride
SDS-PAGE: Sodium Dodecyl Sulfate Polyacrylamide Gel Electrophoresis
shRNA: Small hairpin RNA
siHSF4: Small interfering RNA targeting HSF4
SRA: Sequence Read Archive
ssGSEA: Single-sample gene set enrichment analysis
TCGA: The Cancer Genome Atlas
TNM: Tumour, Node, Metastasis
