# Supplementary material for: Extracellular matrix stiffness regulates colorectal cancer progression via HSF4
Source: J Exp Clin Cancer Res. 2025 Jan 30;44:30. doi: 10.1186/s13046-025-03297-8 (PMC11780783; doi:10.1186/s13046-025-03297-8)

**Extracellular Matrix Stiffness Regulates Colorectal Cancer Progression via HSF4**

Kangtao Wanga, Siyi Ning, Shuai Zhang, Mingming Jiang, Yan Huang, Haiping Pei, Ming Li, Fengbo Tan

## Table of Content

Table S1. List of Antibodies Used in the Study

Figure S1. HSF4 expression is not correlated with collagen levels in CRC.

Figure S2. si-HSF4 #2 demonstrates higher efficiency in suppressing HSF4 expression.

Figure S3. Differential effects of HSF4 knockdown on EMT marker protein expression in SW480 and HCT116 colorectal cancer cell lines under varying substrate stiffness.

Figure S4. HSF4 and increased substrate stiffness promote β-catenin expression and nuclear translocation in HCT-116 cells, facilitating EMT

Figure S5. Overexpression of HSF4 promotes migration and invasion in colorectal cancer cell lines SW480 and HCT116.

Figure S6. Confirmation of LOXL1 overexpression in CCD-18Co cells via fluorescence analysis.

## Table S1: List of Antibodies Used in the Study

| **Antibodies** | **Source** | **Identifier** | **Usage** |
| --- | --- | --- | --- |
| HSF4 antibody | Santa Cruz Biotechnology | sc-398645 | Western Blot; Immunohistochemistry |
| GAPDH polyclonal antibody | Thermo Fisher Scientific | RRID: AB_568552 | Western Blot |
| E-Cadherin (24E10) Rabbit | Cell Signaling Technology | CAT# 3195 | Western Blot |
| N-Cadherin (D4R1H) XP Rabbit | Cell Signaling Technology | CAT# 13116 | Western Blot |
| Vimentin (D21H3) XP Rabbit | Cell Signaling Technology | CAT# 5741 | Western Blot |
| β-Catenin (D10A8) XP Rabbit | Cell Signaling Technology | CAT# 8480 | Western Blot; Immunofluorescence Assay |
| LOXL1 | Servicebio | GB114357;PA5-87701 | Immunohistochemistry |
| α-SMA | Servicebio | GB111364; P62736 | Immunohistochemistry |
| Masson dye solution set | Servicebio | G1006 | Masson Staining |
| HRP conjugated Goat Anti-Rabbit IgG (H+L) | Servicebio | GB23303 | Immunohistochemistry |

## Figure S1. HSF4 expression is not correlated with collagen levels in CRC. (A) Correlation analysis between HSF4 immunohistochemistry (IHC) results and Masson-stained collagen content in 107 patients showed no significant correlation (p > 0.05, R = 0.14). Pearson's correlation was used, with the grey area representing the 95% confidence interval. (B) Violin plot illustrating the distribution of HSF4 IHC results and Masson-stained collagen content in the same cohort, indicating no significant association between HSF4 expression and collagen content in CRC tumours. Independent samples t-test was used for analysis; ns denotes no significances.


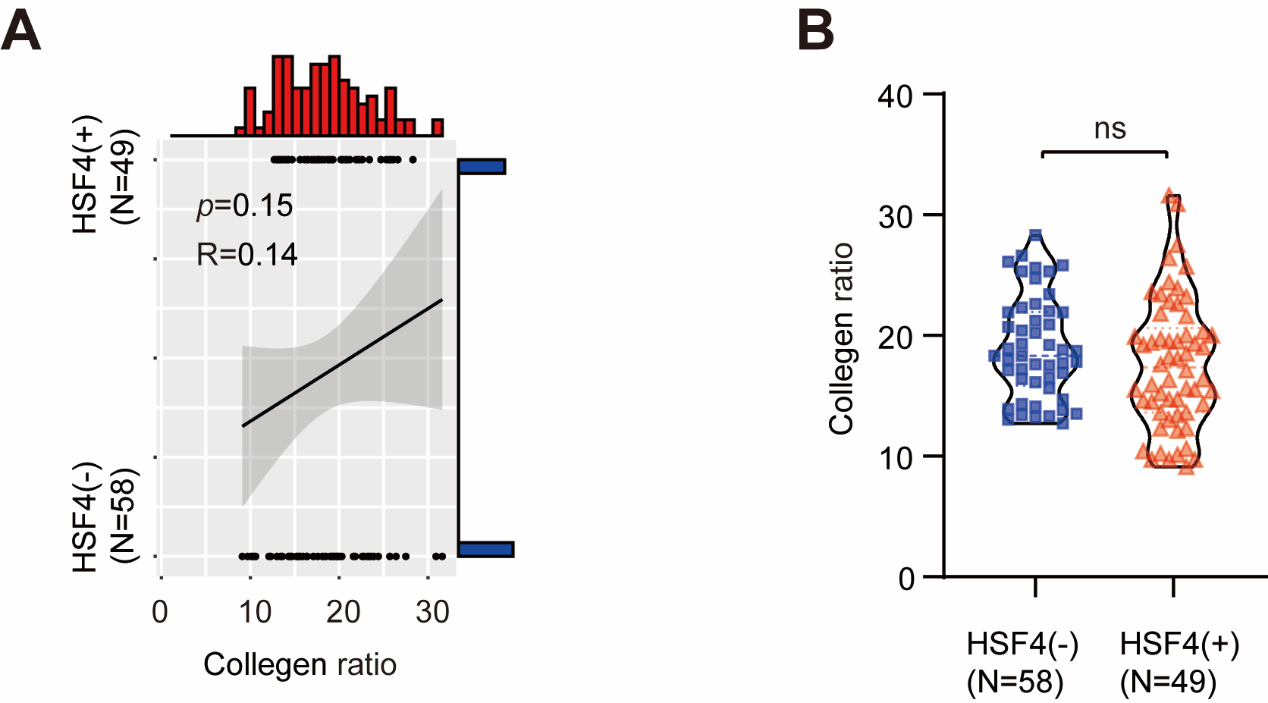


## Figure S2. si-HSF4 #2 demonstrates higher efficiency in suppressing HSF4 expression. Western blot (WB) analysis shows that both si-HSF4 #1 and si-HSF4 #2 significantly inhibit HSF4 expression in SW480 and HCT116 cells, with si-HSF4 #2 exhibiting a more substantial inhibitory effect.


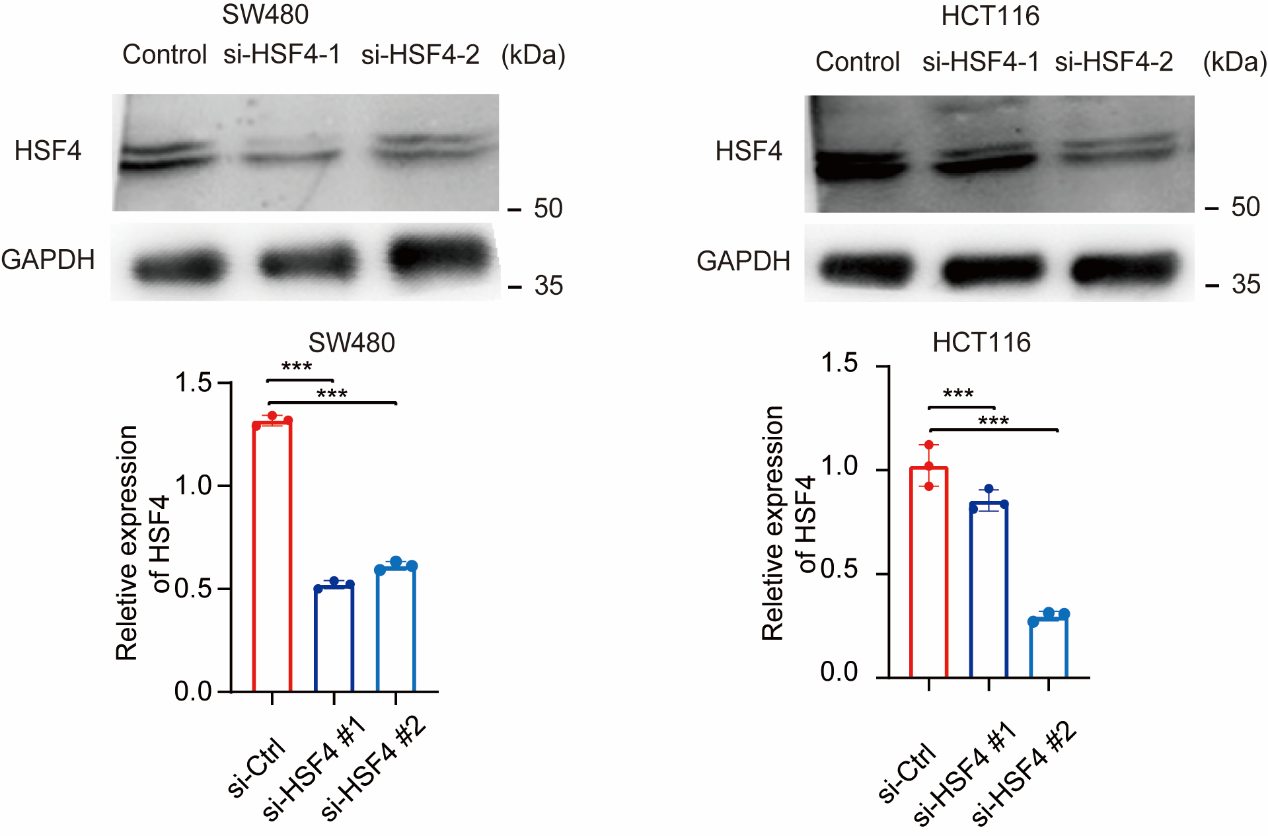


## Figure S3. Differential effects of HSF4 knockdown on EMT marker protein expression in SW480 and HCT116 colorectal cancer cell lines under varying substrate stiffness. (A) Representative Western Blot (WB) analysis of EMT marker proteins (E-Cadherin, N-Cadherin, Vimentin) in SW480 (left panel) and HCT116 (right panel) cells cultured on substrates with varying stiffness (2kPa, 25kPa, 50kPa) following HSF4 knockdown (shHSF4) or in control cells (shCtrl). GAPDH was used as a loading control. (B) Quantification of E-Cadherin protein expression normalized to GAPDH in SW480 (left) and HCT116 (right) cells. HSF4 knockdown significantly increased E-Cadherin expression across different stiffness conditions, with the response more pronounced in SW480 cells. (C) Quantification of N-Cadherin protein expression normalized to GAPDH in SW480 (left) and HCT116 (right) cells. HSF4 knockdown significantly reduced N-Cadherin expression, with SW480 cells showing more consistent suppression across different stiffness levels compared to HCT116 cells, where stiffness had a more substantial modulatory effect. (D) Quantification of Vimentin protein expression normalized to GAPDH in SW480 (left) and HCT116 (right) cells. HSF4 knockdown significantly reduced Vimentin expression in both cell lines, but stiffness had a more pronounced modulatory effect in HCT116 cells. Statistical analysis: Three independent experiments show Data as mean ± SD. Statistical significance was determined using one-way ANOVA followed by Tukey's multiple comparison test (***p < 0.001).

**
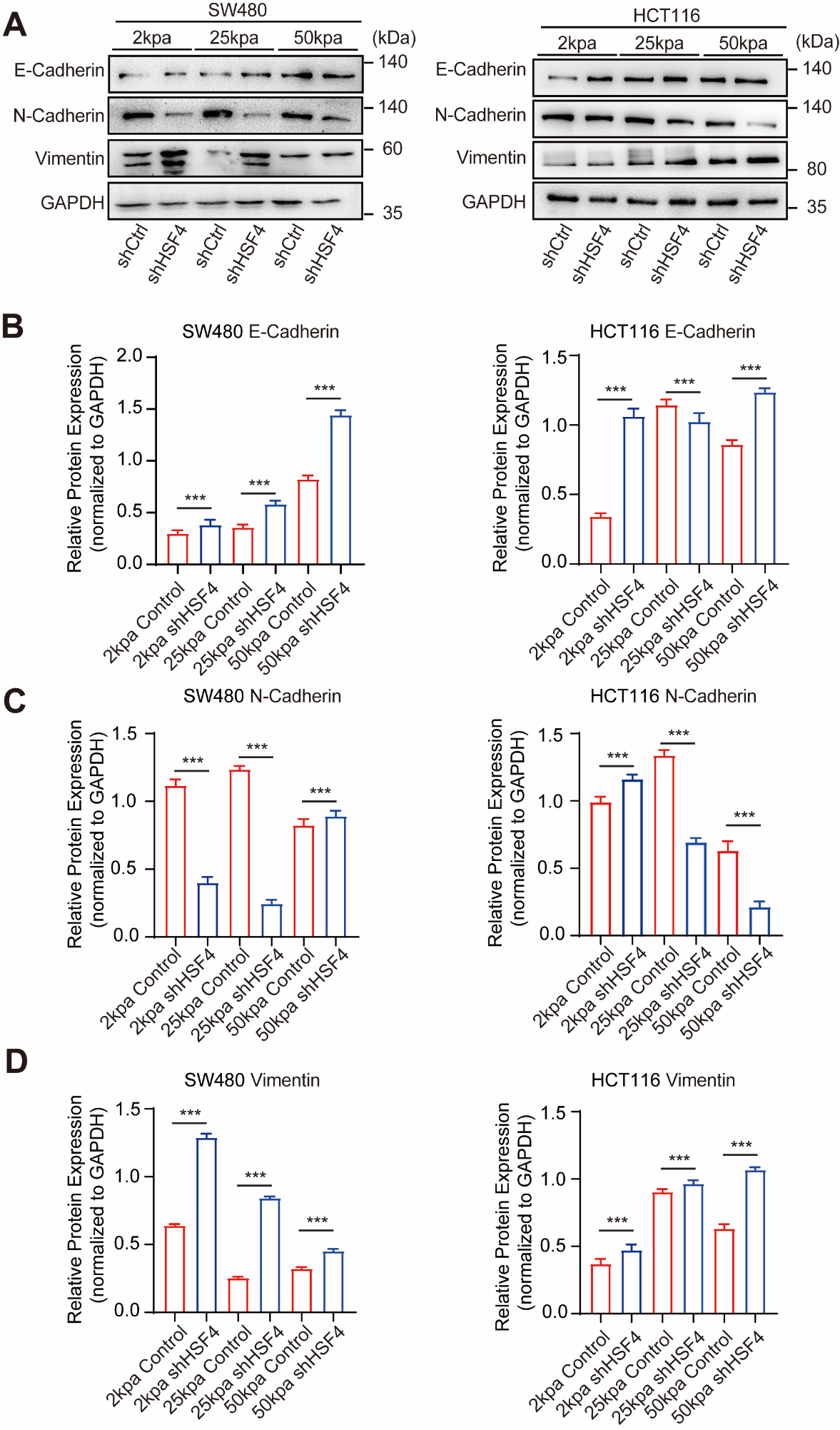
**

## Figure S4. HSF4 and increased substrate stiffness promote β-catenin expression and nuclear translocation in HCT-116 cells, facilitating EMT. Immunofluorescence staining of β-catenin in HCT-116 cells and HSF4 knockdown cells (shHSF4) under three stiffness conditions (2 kPa, 25 kPa, and 50 kPa) demonstrates that both increased stiffness and HSF4 activity lead to elevated β-catenin expression and its nuclear localization, thereby promoting EMT.

**
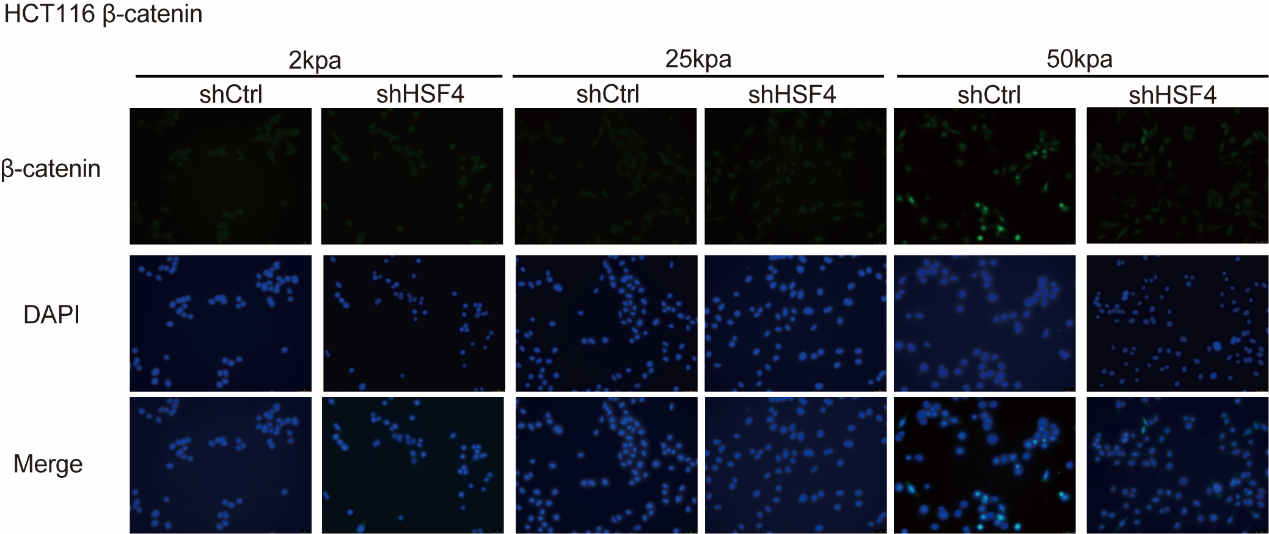
**

## Figure S5. Overexpression of HSF4 promotes migration and invasion in colorectal cancer cell lines SW480 and HCT116. (A) Western blot analysis showing the overexpression of HSF4 in SW480 and HCT116 colorectal cancer cell lines after transfection with the pcDNA-6xHis G-HSF4 plasmid (O/E-HSF4). GAPDH was used as a loading control, and HisG validated the successful transfection of the plasmid. (B) Migration and invasion assays in SW480 cells. Representative images of migrated (upper panel) and invaded (lower panel) cells in the mock and O/E-HSF4 groups are shown. Quantification graphs on the right show a significant increase in migration and invasion in the O/E-HSF4 group compared to the mock group (P < 0.001, unpaired two-tailed Student's t-test). Scale bars: 200 μm. (C) Migration and invasion assays in HCT116 cells. Representative images of migrated (upper panel) and invaded (lower panel) cells in the mock and O/E-HSF4 groups are shown. Quantification graphs on the right show a significant increase in migration and invasion in the O/E-HSF4 group compared to the mock group (P < 0.01, unpaired two-tailed Student's t-test). Scale bars: 200 μm. Statistical analysis: Data are presented as mean ± standard deviation (SD). Significance was calculated using the unpaired two-tailed Student's t-test.


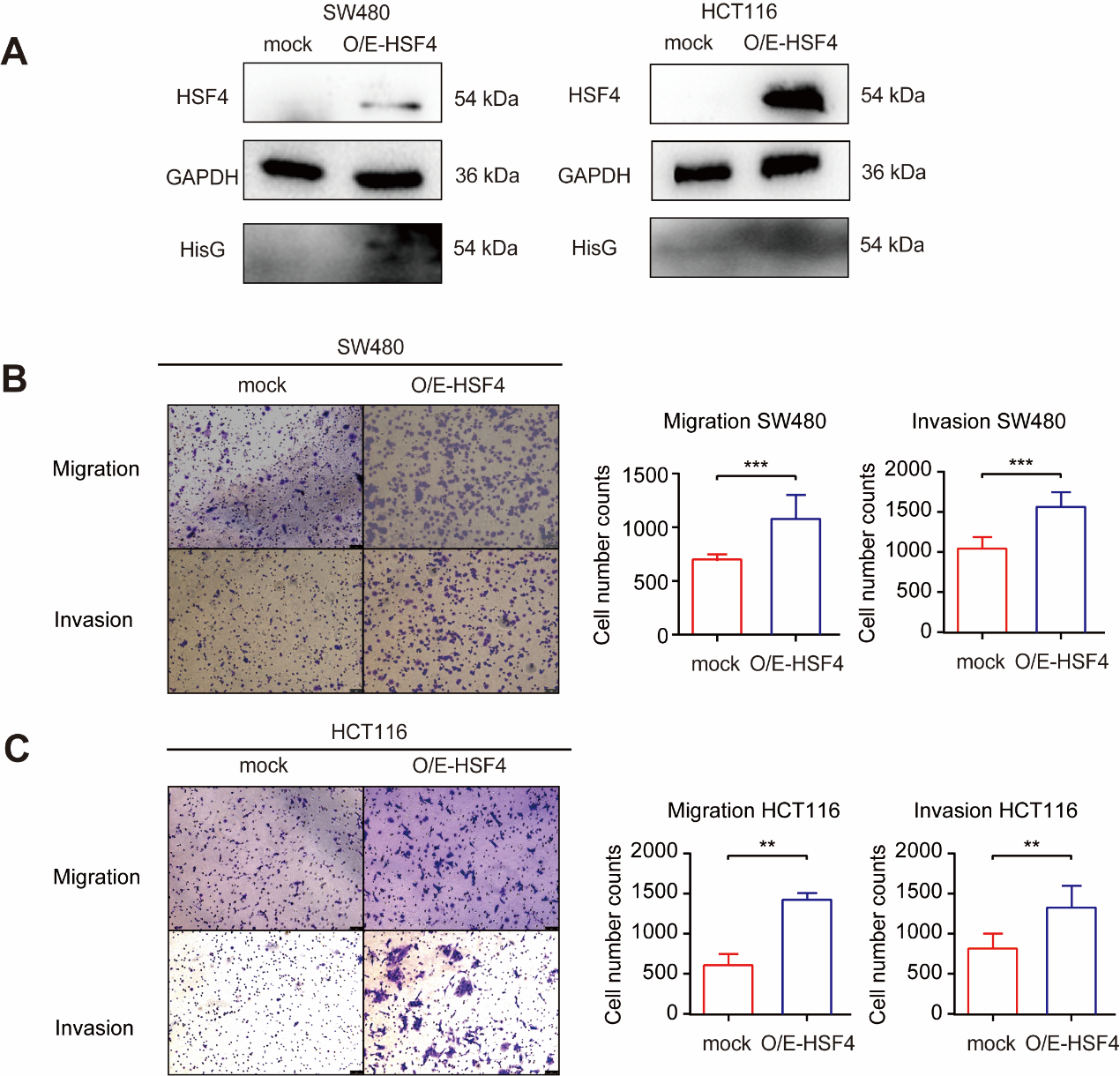


## Figure S6. Confirmation of LOXL1 overexpression in CCD-18Co cells via fluorescence analysis. The left panel shows the light microscopy images of CCD-18Co cells, while the right panel displays green fluorescence, indicating successful overexpression of LOXL1. Scale bars: 200 μm.


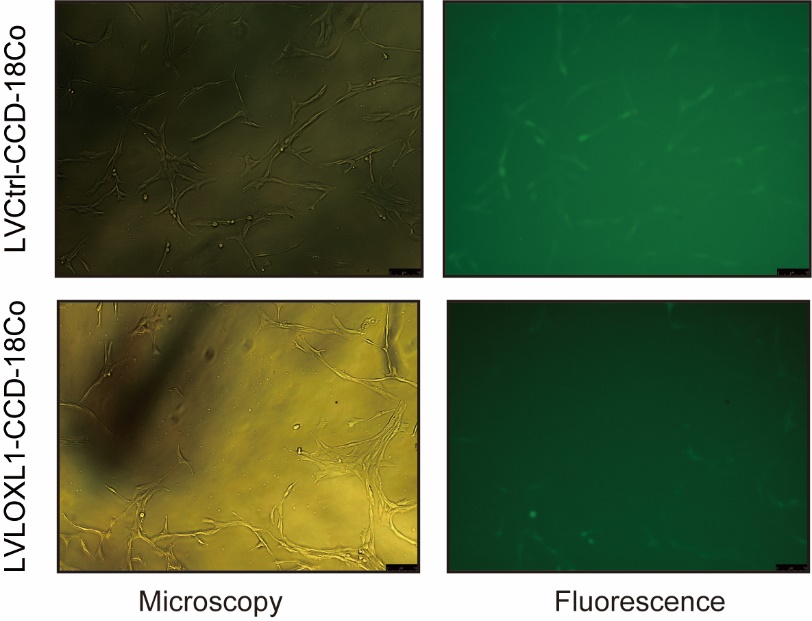

Supplement: Supplementary file 1 — Additional file 1. [file 13046_2025_3297_MOESM1_ESM.docx]
